# Open data on industry payments to healthcare providers reveal potential hidden costs to the public

**DOI:** 10.1038/s41467-019-12317-z

**Published:** 2019-09-20

**Authors:** Jorge Mejia, Amanda Mejia, Franco Pestilli

**Affiliations:** 1 Kelley School of Business, Indiana University, Bloomington, IN 47405 USA; 2 Department of Statistics, Indiana University, Bloomington, IN 47408 USA; 3 Program in Neuroscience, Indiana University, Bloomington, IN 47405 USA; 4 Department of Psychological and Brain Sciences, Indiana University, Bloomington, IN 47405 USA; 5 Program in Cognitive Science, Indiana University, Bloomington, IN 47405 USA

**Keywords:** Health care economics, Health services, Medical ethics, Public health

## Abstract

Healthcare industry players make payments to medical providers for non-research expenses. While these payments may pose conflicts of interest, their relationship with overall healthcare costs remains largely unknown. In this study, we linked Open Payments data on providers’ industry payments with Medicare data on healthcare costs. We investigated 374,766 providers’ industry payments and healthcare costs. We demonstrate that providers receiving higher amounts of industry payments tend to bill higher drug and medical costs. Specifically, we find that a 10% increase in industry payments is associated with 1.3% higher medical and 1.8% higher drug costs. For a typical provider, for example, a 10% or $25 increase in annual industry payments would be associated with approximately $1,100 higher medical costs and $100 higher drug costs. Furthermore, the association between payments and healthcare costs varies markedly across states and correlates with political leaning, being stronger in more conservative states.

## Abstract (web summary)

Industry payments to healthcare providers for non-research costs are common in the US. Linking Medicare and Open Payments data, the authors find that providers receiving more industry payments tend to bill higher drug and medical costs, and this association is stronger in more conservative states.

## Introduction

Human behavior and decision-making are subjective and can be influenced by political, economic, and even social factors, such as norms^1–8^. A global conversation is underway regarding the potential influence of financial players on decision-making related to public policy and social systems. Although much of this conversation has focused on the influence of money in politics, these concerns also extend to other aspects of society. In the United States, healthcare providers, in particular, are allowed to sustain financial relationships with corporations that sell medical devices and pharmaceuticals. At the same time, the United States is also dealing with the highest healthcare costs in the world^9^. Concern has been put forth about the extent and manner of the industry’s influence on the behavior of individual healthcare providers and the implications for healthcare costs^10^. As of today, the full extent of such influence is not well understood.

In 2016, healthcare consumers in the United States paid more for less, spending about twice as much per capita ($10,348) than people in other developed countries, while falling short of those countries in terms of performance measures, such as health outcomes and access^11,12^. In aggregate, healthcare costs amounted to $3.3 trillion or 17.9% of the US Gross Domestic Product (GDP) in 2016 and are expected to grow to almost 20% of GDP by 2026^13^. In response to this growing problem, academics, policymakers, and the general public are striving to understand the drivers of and ultimately reduce healthcare costs^14^.

Two expenditure categories are the primary drivers of healthcare costs in the United States: pharmaceuticals and healthcare service utilization^9^. The US Department of Health and Human Services reported that drug-related costs are among the largest cost drivers in Medicare^13^, accounting for ~17% of total individual healthcare spending ($457B) or $1443 per capita in 2016^9,15^. It has also been noted that pharmaceuticals are a major source of the differences in healthcare costs between the United States and other similar countries^9^. As for utilization of services, it is established that medical providers in the US bill for more medical services and charge a higher cost per service versus those in comparable countries^16^. While previous work has examined these two cost drivers in isolation, the role of pharmaceutical companies in healthcare costs is complex and involves potential conflicts of interest^17^. For example, in the United States, healthcare industry players are allowed to pay for medical providers’ general and research expenditures, and providers are allowed to directly own and invest in the healthcare industry^18,19^. This relationship and its association with healthcare costs are the focus of this article.

To help address concerns about this potential conflict of interest between medical providers and the healthcare industry, the 2010 Affordable Care Act (ACA) established the Open Payments (OP) system. OP aims to provide a more transparent and accountable healthcare system by publishing data on the financial ties between industry and medical providers^20^. As a result of the ACA, medical device corporations (the official name for healthcare industry organizations that include pharmaceutical companies) are required to report payments to all medical providers participating in Medicare^21^. Extant work examining OP data have demonstrated the large scale of the financial ties between pharmaceuticals and medical providers. For example, in 2016, 2005 medical device corporations spent $9.15B collectively on 904,922 physicians across the United States^22^. It is also estimated that US pharmaceuticals spend more on advertising and marketing than on R&D^19,23^.

Substantial research has focused on the impact of industry payments and more generally medical marketing on providers’ attitudes and behavior in the US^24^. For example, higher payments to medical providers by the healthcare industry have been linked to the prescription of brand-name drugs, even when equally effective generic substitutes are available^25–30^. In a more pernicious context, previous work demonstrated a relationship between payments to medical providers from opioid manufacturers and an increase of opioid prescriptions, which may have contributed to the ongoing opioid epidemic^18,31^. These findings have also been documented in the press by Propublica, a nonprofit investigative news organization, in their Dollars for Docs report^32^. Although most of these studies have focused on the healthcare industry’s influence over drug prescriptions^10^, medical spending on services (e.g., procedures, utilization) represents a much larger fraction of overall healthcare costs than drug spending. For example, in the 2016 Medicare data, we report on below, allowed medical costs amounted to over $82B, or over five times the total drug costs of $15B.

There is a paucity of research on the association between industry payments and overall healthcare costs. This may be in part owing to the challenge in linking OP to healthcare data on individual providers, because the National Provider Identifier (NPI) is missing from the OP data set^22,24^. To address this difficulty, we programmatically match over 90% of the NPIs in the 2016 OP data set. We use the NPI-matched OP data for all the analyses in this paper, which we make openly available for the research community to facilitate future research.

The objective of our study is to quantify the relationship between payments to providers from the pharmaceutical industry and healthcare costs. Rather than focusing on specific procedures or drugs, our scope is total Medicare costs, broken down by medical services and drugs. Although our goal is not to establish a direct causal link between industry payments and healthcare costs, we include several important controls to estimate this association robustly. Further, as healthcare delivery varies across US states^33–35^, we also examine the extent to which these financial ties vary geographically and investigate potential political, economic and social drivers of such differences. The results we present below contribute to a better understanding of the pervasive association between industry payments to providers and healthcare costs in the United States.

## Results

### The association between industry payments and medical costs

We present analyses based on the merging of two primary open data sources: the OP system, which contains information on healthcare industry general payments to individual medical providers; and the Medicare Provider Physician and Other Supplier (MPOS) data from the Centers for Medicare and Medicaid Services (CMS), which contains data on providers’ Medicare services and procedures, including the cost (allowed amount) of each procedure. Unlike the CMS data, the OP system does not include the NPI, a unique provider identifier. Without this linking variable, it is difficult to connect the two data sets to study the relationship between industry payments in the OP system and medical costs in the CMS data. To overcome this, we attempted to determine the NPI of providers in the OP system by querying the NPI Public Registry. Based on name and address, we found exact matches for 90.4% of the unique providers appearing in the 2016 OP data, 374,766 of which also appear in the 2016 MPOS data. The linked payment and cost data on these providers form the basis of all subsequent analyses.

**Fig. 1:**
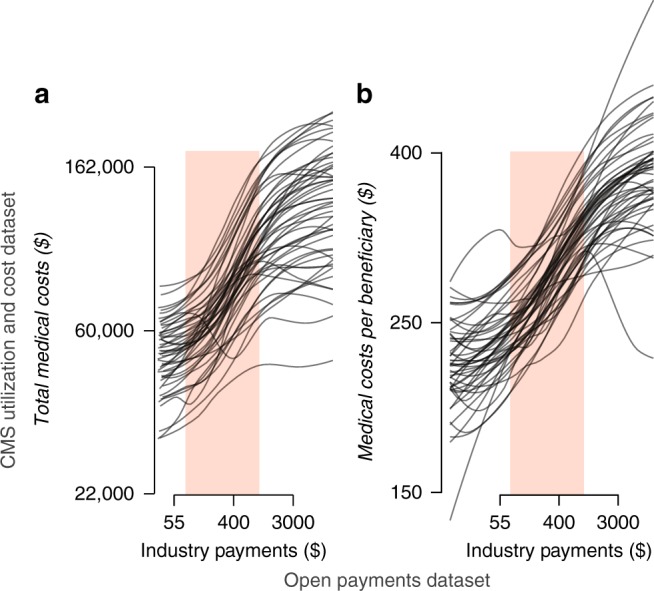
Exploring the relationship between industry payments to providers and medical costs. Each line represents a loess smoother of the scatterplot of the providers within each state (see Methods for details). The shaded bands mark the middle 50% of the distribution of payments. Both axes are displayed on the log scale. The data displayed on the *x* axis comes from the Open Payments data set (openpaymentsdata.cms.gov); the data displayed on the *y* axis comes from the CMS Utilization and Cost data set (cms.gov). See also Supplementary Fig. 1. **a** Total industry payments versus total annual medical (non-drug) costs of each provider. **b** Total industry payments versus average annual medical costs per beneficiary of each provider

Figure 1 displays exploratory plots of the relationship between providers’ industry payments and their annual medical costs, both total (Fig. 1a) and per beneficiary (Fig. 1b). Both axes are displayed on the log scale. As each scatterplot consists of over 300,000 providers, in lieu of individual data points, we display a loess smoother for the scatterplot of providers within each US state (see Methods for details; individual observations are shown in Supplementary Fig. 1). We observed an increasing relationship between payments and costs, with a fairly consistent pattern across states. Although this pattern was nonlinear across the full range of industry payments, it was approximately linear within the middle 50% of the distribution of payments, indicated by the orange shaded bands in Fig. 1.

To quantify the association between providers’ industry payments and medical (e.g., non-drug) costs, we fit a log–log regression model with costs as the response variable (Model 1; see Methods for details). We considered only providers within the middle 50% of the payment distribution (from $77 to $940 in annual industry payments), as the relationship is approximately linear within this range (see Fig. 1). We controlled for state, as we observed baseline differences in costs across states (see Fig. 1), and for the number of beneficiaries served by each provider, as providers serving more beneficiaries tended to receive higher levels of industry investment. We found that a 10% difference in industry payments was associated with ~1.3% higher total medical (non-drug) costs per provider, based on the estimate of *β*_1_ in Model 1 (see Methods section) of 0.131 (95% CI: (0.127, 0.136)). Note that this association is the same when considering providers’ costs per beneficiary as the response variable, as the number of beneficiaries is included as a control in the model.

The association reported above is distinct from that between industry payments and prescribing behavior that has been previously reported^10^. Consistent with these previous reports, we observed an association between industry payments and drug costs, with a 10% difference in payments being associated with an estimated 1.8% higher drug costs (Model 2). To further test the robustness of the new association with medical costs, we re-estimated the association controlling for the provider’s total drug costs (Model 3). In this model, the estimate of *β*_1_ was 0.091 (95% CI: (0.086, 0.095)), so that a 10% difference in industry payments was associated with a 0.91% difference in medical costs, given fixed drug costs. This test served three primary purposes. First, it established that the association between industry payments and medical costs was not spurious or owing to the correlation between providers’ medical and drug costs (Spearman rank correlation = 0.41 among providers with non-zero drug costs). Second, it showed a significant association between industry payments and medical costs above and beyond the association between such payments and drug costs suggested by previous work. Finally, it provided an additional control to establish an association between industry payments and medical costs among physicians that are comparable in terms of several key factors (namely location, size, and drug costs). We note that this robustness test complements, rather than replaces, the original model having a 1.3% effect size, as drug and medical costs tend to increase together in practice^9^.

To put a 1.3% difference in total medical costs in context, consider a typical provider within the middle range of industry payments, with industry payments equal to the median of $250 and total Medicare medical costs equal to the median of $90,646. A 10% difference of $25 in industry payments would then be associated with an expected difference of ~$1,100 in total medical costs. Controlling for drug costs, the expected difference in medical costs would be 0.91%, or $825. Such differences in medical costs are much larger than the differences in industry payments with which they are associated. Therefore, even a small numerical difference in payments is associated with a much larger difference in expected medical costs. Further, although the estimated effect size of 1.8% for drug costs is larger than the 1.3% estimated effect size for medical costs, physicians tend to have much higher medical costs than drug costs. In fact, 61% of the providers in this analysis had no drug costs. Among providers within the middle range of industry payments, the median non-zero drug costs were $5,862, so that a 10% difference in industry payments would be associated with a difference in drug costs of 1.8% or ~$100.

### Geographical variation in the payments–costs association

We investigated how the association between industry payments and medical costs varied geographically within the United States. First, we fit a log–log regression model with a state-specific slope to quantify the association between industry payments and medical costs within each state (Model 4). Indeed, the estimated state-level log–log regression slope varied markedly, ranging from  approximately 0.06 in Maine and Nevada to over 0.22 in Louisiana (Supplementary Table 1). That is, a 10% difference in industry payments to a provider would be associated with an estimated 2.2% difference in medical costs in Louisiana but only a 0.6% difference in Maine or Nevada. We exclude Vermont, which is a clear outlier in terms of both the estimated regression slope of −0.12 and the overall shape of the relationship between payments and medical costs observed in Fig. 1. This may be owing to small sample size compared with the other states (Supplementary Table 1).

**Fig. 2:**
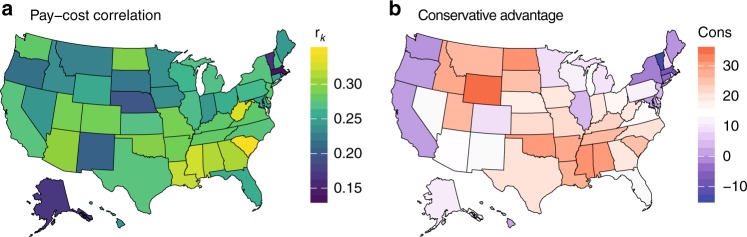
Geographical distribution of the payments–costs association and conservative advantage. **a** Partial Spearman correlation (*r*_*k*_) between industry payments to healthcare providers and their total Medicare medical (non-drug) costs, controlling for the number of beneficiaries per provider, in each state. **b** State-wide conservative advantage as reported by the 2016 Gallup poll on conservative ideology^35^. Among several key political, economic and social factors, conservative advantage was the most strongly correlated with *r*_*k*_

Although the log–log regression slope provides a highly interpretable measure of the association between providers’ industry payments and medical costs, it is limited to describing linear relationships. Therefore, our model focused only on the middle range of payment values (see Fig. 1). To measure the strength of the payment–cost relationship within each state across the entire range of industry payments, we computed the partial Spearman rank correlation coefficient, denoted *r*_*k*_ for state *k*, controlling for the number of beneficiaries of each provider (Supplementary Table 1). Unlike the regression slope, Spearman correlation is appropriate for nonlinear relationships and is invariant to log transformation, and is robust to outliers. Figure 2a displays a map of the *r*_*k*_ values of each state. We observed notable differences between states. States with particularly low *r*_*k*_ values include Vermont, Massachusetts, and Alaska; in these states, there is a positive but relatively weak association between providers’ industry payments and Medicare medical costs. States with the highest *r*_*k*_ values include Louisiana, West Virginia, and South Carolina; in these states, there is a strong positive association between those payments and costs.

### Variance relates to political and population factors

The geographic differences in the payments–costs relationship observed in Fig. 2a suggest potential associations with state-level political, economic, or social factors. To better understand the potential drivers of this geographic variance, we therefore considered several key factors (see Methods section for details on data sources). First, from the American Community Survey, we considered population size (16 years and older), unemployment rate, median household income, high school graduation rate, and percentage of people without health insurance. Second, we considered state-wide differences in the provider market, namely the percentage of medical providers classified as specialists in each state. Third, we considered healthcare quality in each state using the McKinsey & Co. Leading States Health Care Index, a composite score based on access, quality, population health. Finally, we considered a key measure of political leaning, conservative advantage as measured by Gallup, Inc. Among all measures, those that correlated most strongly with *r*_*k*_ of each state were conservative advantage (Pearson correlation 0.50), followed by high school graduation rate (−0.46). Figure 2b displays the conservative advantage by state; indeed, we observe some similarities with geographical patterns of the strength of the industry payments-medical costs association displayed in Fig. 2a.

To jointly examine potential drivers of the geographical variation in the strength of the payments–cost association, we turned to a model-based approach. Specifically, we fit a multiple linear regression model of *r*_*k*_ against all of the measures listed above, which controls for possible confounding among the various political, economic, and social factors (Model 5). Note that this model is not concerned with the amount of medical costs or industry payments, but rather the strength of the association between providers’ industry payments and medical costs (i.e., *r*_*k*_) after controlling for other key provider-level factors.

**Fig. 3:**
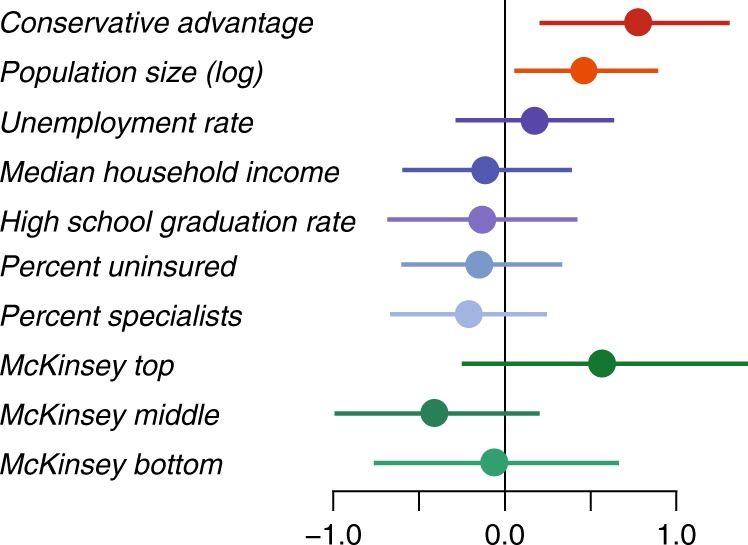
Coefficient estimates for regression of *r*_*k*_ against key state-level factors (Model 5). Conservative advantage and population size (warm colors) were significantly and positively associated with *r*_*k*_ at the 0.01 level. None of the other factors (socioeconomic in blues; health in greens) were significantly associated with *r*_*k*_. Values close to ±1 indicate a strong relationship with *r*_*k*_ for continuous variables. Error bars show Normal two-sided 99% confidence intervals

Prior to model fitting, all continuous variables were standardized to allow for comparability of coefficients across the predictor variables and to provide an interpretable scale, where values close to ±1 indicate a strong association with *r*_*k*_. The McKinsey rankings were grouped into upper, middle and lower thirds, and the model includes a coefficient associated with each group. The coefficient estimates and normal 99% confidence intervals for each predictor variable are displayed in Fig. 3; estimates and *p* values based on a two-sided *t* test against the null hypothesis that each coefficient equals zero are reported in Supplementary Table 2. Conservative advantage showed the strongest association with *r*_*k*_ among all continuous variables (0.706; *p* = 0.001), indicating a strong association between a state’s conservative leaning and the strength of the payments–costs relationship after controlling for other important state-level factors. Besides this political measure, no other economic or social factors were significantly associated with *r*_*k*_, with the exception of log population size (0.460; *p* = 0.006). The McKinsey & Co. health rankings were not statistically significant when considered jointly using an *F* test (degrees of freedom = 43 and 3) against the null hypothesis that all three coefficients are zero (*F* = 1.903; *p* = 0.145), though individually some groups showed effects trending toward significance (see Supplementary Table 2). Therefore, our findings suggest that larger and more-conservative states tended to have stronger-than-expected associations between industry payments and medical costs of individual providers.

## Discussion

Much of the research on healthcare costs has focused on the potential for cost reduction through changes in access, utilization, and efficiency^16,36,37^. By contrast, our study considers how the growing total cost of healthcare may be related to payments to providers from the healthcare industry. We identified an association between industry payments and medical costs, in addition to an association between such payments and drug costs consistent with previously reports. Specifically, we found that for a typical provider, a $25 difference in payments was associated with a difference of $1,100 in annual Medicare medical costs and of $100 in annual Medicare drug costs. Moreover, the strength of the association between payments and medical costs varied geographically and was associated with state-level political ideology and population size; more conservative and larger states tended to have stronger associations between industry payments and medical costs. Although extant work had established that medical providers’ political ideology may influence patient care^38^, our study is novel in investigating how a state’s political affiliation may be related to the interface between medical providers and the healthcare industry.

In response to potential conflicts of interest that may harm the public, the common response from academics and policymakers is calls for increased transparency^39^. Most notably, the 2010 US Affordable Care Act added a number of targeted provisions to increase transparency in the healthcare system. Indeed, our study was possible because of the open data made available through such provisions, specifically the Sunshine laws, which created the OP system to make financial disclosures between suppliers (i.e., pharmaceuticals) and providers (i.e., physicians) mandatory. These data have allowed researchers to begin measuring and tracking the financial ties between the healthcare industry and providers. Despite this new transparency, however, these ties remain substantial and do not seem to be slowing down. In fact, reported payments more than doubled between 2013 and 2016, from $4.33 billion to $8.81 billion, according to OP. Furthermore, a recent survey showed that very few Americans were aware that this information is publicly available, and only 5% knew whether their doctor had received payments^40^. Therefore, transparency alone, without effective communication to consumers or adoption of evidence-based policies, has not been sufficient to curtail the payments of large medical corporations to medical providers. These limits of transparency have been noted previously, most notably by Weil et al.^39^, who concluded, “Consumers may not understand or be interested in new information, may not have the capacity to choose when given information, or may not act in accord with policymakers’ aims”.

To make actionable use of the transparency provided by the OP system, we believe more work is needed to help patients access and interpret the data. As a 2017 JAMA article argued, “Transparency regarding public disclosure of payments to physicians from industry, and the relationship between payments and prescribing is still in its infancy. Going forward, as additional data become available, it will be imperative to continue improving the tools available for consumers and provide information and context on how to use them”^32^. Several possible strategies for improved use of OP data to empower healthcare consumers have been suggested, including flagging unusually high payments^21^, going beyond disclosure to incentives^41^, educating patients on how to interpret OP data^42^, and expanding the reach of the OP system^43^. A suggestion we would like to put forth is to provide OP information to the public at the point of service, a strategy that has been utilized effectively in other contexts, including restaurant hygiene, auto safety ratings and appliance energy efficiency metrics^39^. For example, restaurant hygiene scorecards have led to important decreases in foodborne illness^44^. By giving the public digestible information on physicians’ industry ties at the point of service, patients would be put in charge of deciding how much they value the independence of their providers from the healthcare industry. An important first step, however, is to investigate how consumers can accurately gauge and interpret the information in the OP system and how they may act in response.

We hope our study will spur future research on the implications of industry payments in the healthcare system, with the ultimate goal of impacting policy making. Such research is currently limited by the exclusion of the NPI from the OP system. Inclusion of the NPI in OP would allow researchers to link data on industry payments directly to the other data sets provided by CMS. Although we were able to match 90.4% of the providers in the 2016 OP data programmatically, the lack of a complete linkage introduces the possibility of selection bias. Such linkage is also time-consuming and requires substantial effort and expertize. This is a fundamental limitation of the OP system in its current state. Interestingly, this problem was foreseen and reported on prior to the rollout of OP in 2011^45^.

This study is subject to several other limitations. We focused our analysis on the category of general payments in the OP data set and did not study payments to providers for research purposes. Future studies should focus on understanding the differences between general payments and research payments in terms of their relationships with healthcare costs. In addition, this study was based on Medicare data and does not consider non-Medicare healthcare costs. Finally, our analysis is subject to the limitations of observational studies, namely that it does not establish a causal effect of industry payments on healthcare costs. Given the nature of the data, previous studies investigating the relationship between industry ties and prescribing behavior have also been limited to correlational analyses^32^. Future research is needed to determine the causal mechanisms relating industry contributions to costs.

In the context of our analysis, one possible alternative explanation of the observed association between total industry payments and total healthcare costs is that of reverse causality. That is, providers with high levels of spending may be targeted by the medical device industry as most likely to result in a return on investment. Yet, this suggests that these corporations expect that on average, providers receiving payments will in turn increase spending in ways that will benefit those corporations. Disentangling such a causal feedback loop would require more granular and detailed data on payments and healthcare expenditures than is currently available to the public.

Although establishing causality remains a worthy goal and may be ultimately more effective in provoking changes to policy, public opinion, and provider behavior, to date this has proven elusive. Yet, some have contended that establishing causality may not be a necessary goal. Steinbrook argued that the financial relationships between physicians and the industry, resulting from payments are problematic in and of themselves, stating that “The American Medical Association’s Code of Medical Ethics is clear that ‘[g]ifts to physicians from industry create conditions that carry the risk of subtly biasing—or being perceived to bias—professional judgment in the care of patients.”^22^.

## Methods

### Data sources

The data sources used in the study included the Open Payments (OP) from the Centers for Medicare and Medicaid Services (CMS) (Data set 1), the Medicare Provider Utilization and Payment Data from CMS (Data set 2), the NPI Public Registry from CMS (Data set 3), the Gallup poll on conservative ideology^35^ (Data set 4), and data on state-level economic and social factors compiled from several sources (Data set 5). Data set 1 includes information from manufacturers of drugs and devices (i.e., the healthcare industry) on payments they make to medical providers participating in Medicare. The linkage to Medicare provider utilization and costs in Data set 2 enriches the data and enables us to investigate the effect industry payments may have on healthcare utilization and cost. Linkage with measures of political ideology in Data set 4 allow us to capture how state-level differences in political ideology may shift the relationship between industry payments and provider utilization. Finally, Data set 5 provides state-level controls, such as population and income. All of the data used for both the provider- and state-level analyses are cross-sectional.

Data set 1: Industry Payments. The Open Payments system (https://openpaymentsdata.cms.gov/) requires manufacturers and group purchasing organizations (GPOs) to disclose payments and other transfers of value to medical providers and hospitals every year. One of the stated objectives of the program is that it “helps to identify relationships that can both lead to the development of beneficial new technologies and wasteful healthcare spending”. Although OP is the only national data set of its type linking the industry and medical providers, it does not make any claims on whether the financial relationships are beneficial or whether they cause conflicts of interest. We focus on general payments (i.e., non-research related) for the analyses in the paper.

Data set 2: Resource Utilization and Costs of Care. We used the Medicare Provider Utilization and Payment data (https://www.cms.gov) to examine the costs of care based on Medicare payment amounts. Specifically, we used the Medicare Provider Physician and Other Supplier (MPOS) data, which include information on services and procedures provided to Medicare beneficiaries by individual medical providers. The MPOS contains information on utilization, payment (allowed amount), and submitted charges for each provider, identified with a unique National Provider Identifier (NPI). We examined two types of cost: allowed medical services and allowed prescription drugs for 2016, which is the most recent year available. The 2016 MPOS data include 991,362 individual medical providers performing 2.33 billion individual services, which adds to $107.05 billion in Medicare allowed amounts.

Data set 3: NPI Public Registry. The OP and MPOS data sets both contain identity information about the individual providers, such as the provider name, geographical location, and gender. However, there is no direct link between the data sets, as unlike MPOS, OP does not contain the NPI of each provider. Using the NPI Public Registry from CMS (https://npiregistry.cms.hhs.gov), we queried each provider present in OP to obtain the NPI, which we then linked to the MPOS data set. This allowed us to aggregate data from the OP and MPOS data sets at the individual provider level. Of the 636,871 unique providers appearing in the 2016 OP data set, we found exact matches for 576,144 (90.4%), whose collective payments represented 91.8% of the total 2016 general payments. The matched and unmatched providers did not differ substantially in terms of the amount or number of payments, or in the types of providers represented. Of the matched providers, 374,766 also appeared in the MPOS 2016 data set. This is the set of providers we used for the main analysis of the study.

Data set 4: Gallup Poll on Conservative Ideology. Data pertaining to political orientation were obtained from a poll by Gallup on conservative ideology^35^. These data were based on telephone interviews conducted between January 2 and December 30, 2016, on a sample of 177,788 adults (aged 18 or older) living within the 50 US states and the District of Columbia. The data contains a measure of conservative ideology for each state.

Data set 5: State-level control variables. To control for state-level economic and social factors, we used several data sets: the 2016 American Community Survey (ACS) by the US Census Bureau, an annual survey providing social, economic, housing, and demographic information about US population^46^; the McKinsey & Co. Leading States Health Care Index, a composite score based on access, quality, population health^34^; and the KFF Professionally Active Specialist Physicians by Field data set for 2016, which contains the number of medical providers and specialists by state^47^.

### Statistical analyses

Two primary analyses were carried out during this study. First, we investigated the relationship between industry payments to healthcare providers (i.e, physicians) and the cost of services and drugs billed to Medicare by these providers. Second, we assessed how this relationship varied geographically across the US states and investigated potential drivers of this difference related to state-level political, economic, and social measures. All analysis code is made available for reuse.

We first performed a series of exploratory analyses to examine the shape of the data distribution across the linked individual healthcare providers. We computed skewness and Pearson’s measure of kurtosis for both medical costs and industry payments. The skewness and kurtosis of a Normal distribution are 0 and 3, respectively. The distribution of medical costs had skewness of 8.8 and kurtosis of 214, whereas the distribution of payments had skewness of 179 and kurtosis of 43,139, indicating that both distributions were highly right-skewed. After log transformation, the medical costs distribution had skewness of −0.4 and kurtosis of 3.1, and the payments distribution had skewness of 0.6 and kurtosis of 3.5, indicating that both distributions were approximately log-normal before transformation. Given the extreme right-skewed distribution of both medical costs and industry payments, we performed all analyses on log-transformed data.

### Quantifying the payments–costs relationship

To visually investigate the relationship between industry payments and medical costs, given the large number of observations in our data set we performed loess smoothing of the scatterplot of log payments versus log medical costs (both total and per beneficiary) within each state (Fig. 1). The curves for each state were obtained using stat_smooth of the ggplot2 R package (https://ggplot2.tidyverse.org/) using the default parameter values and methods of the R loess function, including a span of 0.75 (meaning that 75% of all the observations were used in each model) and degree of 2 for the local polynomial regression models. Both plots displayed in Fig. 1 were based on 322,109 providers with both medical costs and industry payments observed.

We observed a consistent nonlinear increasing relationship between payments and medical costs, with an approximately linear segment between the 25th quantile ($77) and 75th quantile ($940) of industry payments, indicated by the shaded band in Fig. 1. To quantify the relationship between payments and medical costs within this range, we fit a linear regression model relating log medical costs to log payments with a different intercept for each state and controlling for the number of beneficiaries serviced by each provider. Specifically, for provider *i* belonging to state *k*_*i*_, we related medical costs (*M*_*i*_) to industry payments (*P*_i_) and number of beneficiaries (*B*_*i*_) with the model

$${\hbox{Model 1:}}\ \log \left( {M_i} \right) = {\beta} _{0k_i} + {\beta} _1\log \left( {P_i} \right) + \beta _2\log \left( {B_i} \right) + {\it{\epsilon }}_i,{\it{\epsilon }}_i {\sim} N(0,{\sigma}^2)$$

That is, we assume that *M*_*i*_ is log-normally distributed with mean *μ*_*i*_ and variance *σ*^2^, where *μ*_*i*_ = E[log(*M*_*i*_)] = $\beta_{0k_i}$ + *β*_1_log(*P*_i_) + *β*_2_log(*B*_*i*_). Thus, E[*M*_*i*_]  = exp(*μ*_*i*_ + *σ*^2^/2) = exp($\beta_{0k_i}$)${P_i}^{\beta_1}\ {B_i}^{\beta_2}$ exp(σ^2^/2). Note that the relative expected medical costs given a 1% change in payments, holding number of beneficiaries and state constant, is $1.01^{\beta_1}$ (which is approximately equal to 1 + *β*_1_/100 for moderate values of *β*_1_, e.g., between −5 and 5). Therefore, the interpretation of the coefficient *β*_1_ in this model is the percentage difference in expected medical costs associated with a 1% difference in industry payments. To fit Model 1, we utilized data from 159,389 providers within the middle range of industry payments with observed medical costs.

We also evaluated two alternative models to assess the robustness of our main model examining the association between medical costs and industry payments. First, we fit a model using drug costs, rather than medical costs, as the response variable. To fit this model, we utilized data from the 61,574 providers within the middle range of industry payments with observed, non-zero drug spending. Letting *D*_*i*_ be the drug costs for provider *i*, this model is given by:

$${\hbox{Model 2:}}\ \log \left( {D_i} \right) = \beta _{0k_i} + \beta _1\log \left( {P_i} \right) + \beta _2\log \left( {B_i} \right) + {\it{\epsilon }}_i,{\it{\epsilon }}_i \sim N(0,\sigma^2),$$

Second, we fit a model similar to Model 1, but including drug costs as an explanatory variable. To fit this model, we utilized data from the 159,389 providers within the middle range of industry payments with observed medical and drug costs. This model is given by:

$${\hskip-13pt}{\hbox{Model 3:}}\ {\mathrm{log}}(M_i) = \beta _{0k_i} + \beta _1 {\mathrm{log}}(P_i) + \beta _2 {\mathrm{log}}(B_i) + \beta _3 {\mathrm{log}}(D_i) + {\it{\epsilon }}_i,{\it{\epsilon }}_i \sim N(0,\sigma^2).$$

In all three models, ***β***_1_ is the coefficient of interest, with the following interpretations:

In Model 1, ***β***_1_ is estimated to be 0.131 (95% CI: (0.127, 0.136)) and represents the percentage difference in expected medical costs associated with a 1% difference in industry payments, given a fixed state and number of beneficiaries.

In Model 2, ***β***_1_ is estimated to be 0.180 (95% CI: (0.156, 0.202)) and represents the percentage difference in expected drug costs associated with a 1% difference in industry payments, given a fixed state and number of beneficiaries.

In Model 3, ***β***_1_ is estimated to be 0.091 (95% CI: (0.086, 0.096)) and represents the percentage difference in expected medical costs associated with a 1% difference in industry payments, given a fixed state, number of beneficiaries, and level of drug costs.

### State-level differences in the payments–costs relationship

To better understand how the relationship between industry payments and medical expenses varies by state, we refit Model 1 within each state. Again considering the middle 50% of industry payments, for observation *i* belonging to state *k*_*i*_, we fit the model

$${\hbox{Model 4:}}\ {\mathrm{log}}(M_i) = \beta _{0k_i} + \beta _{1k_i}\log \left( {P_i} \right) + \beta _{2k_i}\log \left( {B_i} \right) + {\it{\epsilon }}_i,{\it{\epsilon }}_i \sim N(0,\sigma_k^2).$$

The interpretation of $\beta_{1k_i}$ in Model 4 is similar to that of *β*_1_ in Model 1, but is specific to providers in state *k*_*i*_. Estimates and Normal 95% confidence intervals for the *β*_1*k*_ for each state *k* are given in Supplementary Table 1.

The log–log regression slope provides a highly interpretable measure of association between industry payments and medical costs and, importantly, allows us to estimate the percent difference in medical costs associated with a percent difference industry payments. However, it has two drawbacks. First, it is limited to the middle 50% of industry payment values, as the relationship is more linear within this range than across the entire range of payment levels (Fig. 1). Second, even within this range the relationship between payments and medical costs is only approximately linear. Therefore, to measure the overall strength of the payment–cost relationship across the entire range of industry payments within each state, we turned to Spearman correlation. Specifically, we computed the partial Spearman correlation between payments and medical costs, controlling for the number of providers, denoted *r*_*k*_ for state *k*. The *r*_*k*_ values for each state are given in Supplementary Table 1. Note that Spearman rank correlation is appropriate for nonlinear relationships and is invariant to log transformation and any other monotonic transformations. Therefore, it is an apt measure to quantify the overall strength of the payments–costs relationship within each state.

### Geographical variation in the payments–costs association

Figure 2 displays a map of the *r*_*k*_ values of each state, showing differences in the strength of the correlation between industry payments and medical costs of providers. We then investigated how state-level political, economic, and social factors may help explain these differences. We considered the following measures: conservative advantage, population size 16 years and older (log scale), unemployment rate, median household income, high school graduation rate, percentage of people without health insurance, percentage of medical providers classified as specialists, and the McKinsey Leading States Health Care Index, grouped into thirds.

To control for possible confounding factors among the various political, economic and social factors, we fit a multiple linear regression model of *r*_*k*_ against all of the measures listed above. Note that we fit Model 5 using *r*_*k*_ on its original scale rather than Fisher transforming the values, as they are all small to moderate and therefore virtually unchanged by transformation. All continuous variables including *r*_*k*_ were centered and scaled; the coefficients therefore represent the expected standard deviation increase in *r*_*k*_ associated with a 1 s.d. increase in each predictor variable, holding the other variables constant. The McKinsey index was grouped into thirds (top, middle, and bottom) and indicator variables for each group were included in the model, so that the coefficients indicate the expected value of *r*_*k*_ after centering and scaling associated with belonging to each group, given mean values for each continuous variable. Specifically, we fit the following model:

$${\mathrm{Model}}\ 5:\ r_k =	 \ {\beta} _1{\mathrm{Cons}}_k + {\beta} _2{\mathrm{log}}({\mathrm{Pop}}_k) + {\beta} _3{\mathrm{Unemp}}_k \\ 	+ \, {\beta} _4{\mathrm{Inc}}_k + {\beta} _5{\mathrm{HS}}_k + {\beta} _6{\mathrm{Unins}}_k + {\beta} _7{\mathrm{Spec}}_k \\ 	+ \mathop {\sum}\limits_{g = 1}^3 {{\alpha} _g{\mathrm{Health}}_{kg} + {\it{\epsilon }}_k,{\it{\epsilon }}_k {\sim} N(0,\tau^2)}$$

where for state *k*, Cons_*k*_ is conservative advantage, Pop_*k*_ is population size, Unemp_*k*_ is unemployment rate, Inc_*k*_ is median household income, HS_*k*_ is high school graduation rate, Unins_*k*_ is percentage without health insurance, Spec_*k*_ is the percentage of specialist medical providers, and Health_*kg*_ is an indicator variable equal to 1 if the state belongs to the upper (*g* = 1), middle (*g* = 2) or lower (*g* = 3) third of states in terms of health, according to the McKinsey rankings. The coefficient estimates and *p* values based on a two-sided *t* test against the null hypothesis that each coefficient equals zero for Model 5 are given in Supplementary Table 2; coefficient estimates and Normal 99% confidence intervals are displayed in Fig. 3.

All statistical analyses were conducted in R version 3.5.1. The Institutional Review Board at Indiana University exempted this study for approval because all patients in the CMS databases had been de-identified.

### Reporting summary

Further information on research design is available in the Nature Research Reporting Summary linked to this article.

## Supplementary information

Supplementary Information

Peer Review File

Reporting Summary

## Data Availability

Data utilized for this study are available at https://osf.io/y9ge4/.

## Code availability

The custom scripts utilized for this study, including all the statistical analyses, are available at https://osf.io/y9ge4/.
