# Supplementary material for: Open data on industry payments to healthcare providers reveal potential hidden costs to the public
Source: Nat Commun. 2019 Sep 20;10:4314. doi: 10.1038/s41467-019-12317-z (PMC6754508; doi:10.1038/s41467-019-12317-z)
Supplement: Supplementary file 1 — Supplementary Information [file 41467_2019_12317_MOESM1_ESM.pdf]

Supplementary information

Open data on industry payments to healthcare providers reveals potential hidden costs to the public

Mejia et al.

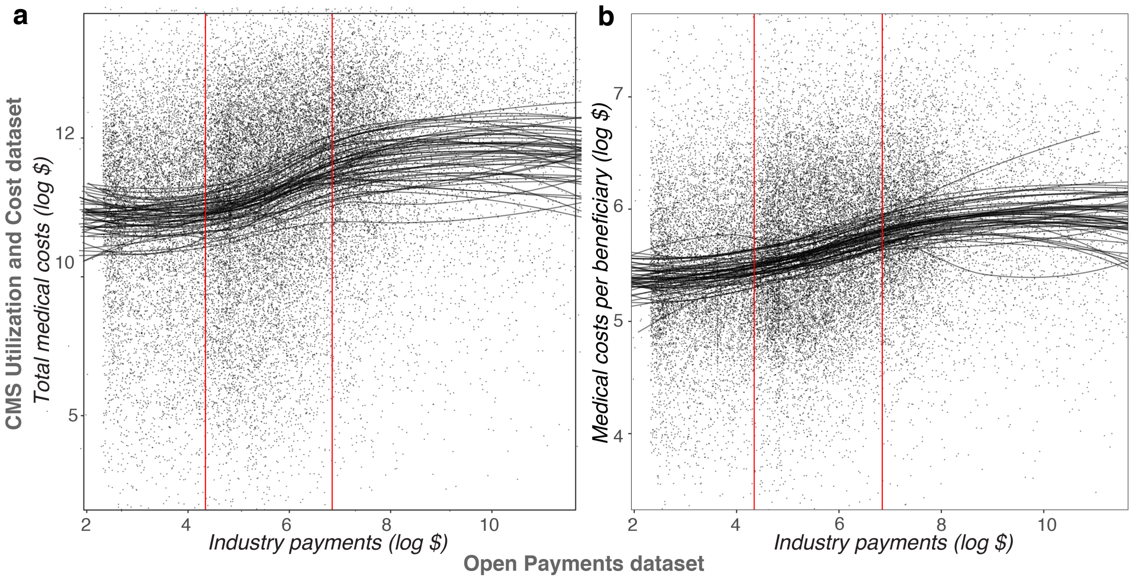

Supplementary Figure 1. The relationship between industry payments to providers and medical costs. Each dot represents an individual provider (dot-cloud uniformly down sampled to 10% to avoid over plotting), and each line is a loess smoother of the scatterplot of all the providers within each state. Loess curves for each state were calculated as in Figure 1, detailed in the Methods section, and are based on 322,109 providers with both industry payments and medical costs observed. The red lines indicates the middle 50% range of the distribution of payments. Both the x- and y-axes are truncated at the 1st and 99th quantiles for visualization purposes. **a.** Total industry payments versus total medical costs of each provider. **b.** Total industry payments versus average medical costs per beneficiary of each provider.

State	n	Log-Log Regression Slope (95% CI)	Partial Spearman Correlation (r_k)	Conservative Advantage
**VT**	177	-0.09 (-0.29, 0.10)	0.16	-14
**MA**	6852	0.12 (0.09, 0.15)	0.13	-8
**CT**	4718	0.09 (0.05, 0.12)	0.25	-4
**NY**	22079	0.14 (0.12, 0.16)	0.27	-2
**ME**	1067	0.07 (-0.02, 0.16)	0.24	0
**WA**	5008	0.13 (0.09, 0.17)	0.28	0
**CA**	28227	0.17 (0.16, 0.19)	0.27	1
**MD**	6864	0.11 (0.08, 0.15)	0.25	1
**OR**	2713	0.13 (0.07, 0.18)	0.22	1
**HI**	1165	0.16 (0.09, 0.24)	0.24	2
**IL**	12674	0.11 (0.09, 0.14)	0.27	4
**NJ**	11802	0.12 (0.09, 0.14)	0.28	4
**RI**	1101	0.10 (0.03, 0.18)	0.19	4
**DE**	1189	0.10 (0.03, 0.18)	0.27	7
**CO**	4540	0.13 (0.09, 0.17)	0.29	9
**MN**	2906	0.08 (0.02, 0.14)	0.24	9
**NH**	1215	0.17 (0.09, 0.24)	0.27	9
**MI**	11122	0.15 (0.13, 0.18)	0.27	10
**AK**	518	0.10 (-0.02, 0.22)	0.17	11
**PA**	16003	0.12 (0.10, 0.14)	0.26	12
**WI**	3945	0.13 (0.09, 0.17)	0.26	12
**FL**	22586	0.12 (0.10, 0.13)	0.26	13
**NV**	2413	0.05 (0.00, 0.11)	0.24	13
**NM**	1461	0.15 (0.08, 0.21)	0.20	13
**AZ**	6239	0.10 (0.07, 0.14)	0.30	14
**VA**	7787	0.13 (0.10, 0.16)	0.28	15
**OH**	13060	0.13 (0.10, 0.15)	0.26	16
**NE**	2006	0.11 (0.06, 0.16)	0.20	18
**GA**	9269	0.14 (0.11, 0.17)	0.31	19
**IN**	7175	0.12 (0.09, 0.15)	0.24	19
**IA**	2683	0.08 (0.04, 0.13)	0.24	19
**WV**	2016	0.17 (0.10, 0.23)	0.33	19
**NC**	10275	0.11 (0.09, 0.14)	0.27	20
**TX**	22994	0.14 (0.12, 0.16)	0.27	20
**KS**	2925	0.17 (0.12, 0.22)	0.27	21
**MO**	6581	0.08 (0.04, 0.11)	0.29	21
**KY**	4941	0.14 (0.11, 0.18)	0.28	22
**SC**	5109	0.18 (0.14, 0.22)	0.35	25
**SD**	915	0.07 (0.00, 0.15)	0.22	25
**MT**	845	0.14 (0.05, 0.24)	0.23	26
**TN**	7026	0.11 (0.08, 0.14)	0.28	26
**UT**	2189	0.11 (0.05, 0.16)	0.29	26
**LA**	5336	0.22 (0.18, 0.25)	0.32	27
**AR**	2844	0.13 (0.09, 0.18)	0.29	28
**ID**	1340	0.11 (0.04, 0.19)	0.24	28
**AL**	5109	0.13 (0.09, 0.16)	0.31	30
**OK**	3976	0.14 (0.10, 0.18)	0.29	30
**MS**	2698	0.15 (0.11, 0.20)	0.32	31
**ND**	711	0.16 (0.05, 0.26)	0.29	31
**WY**	438	0.17 (0.03, 0.30)	0.25	35

Supplementary Table 1. State-level measures of association between industry payments and medical costs and of political leaning. The regression slope and partial Spearman correlation are estimated as described in the Methods. The interpretation of the estimated regression slope is the expected percentage difference in medical costs for a 1-percent difference in industry payments, controlling for state and number of beneficiaries.

Measure	Coefficient Estimate	Two-sided *t*-Test Test statistic (p-value)
**Conservative Advantage**	**0.706**	**3.600 (p = 0.001)**
**Population Size (log)**	**0.460**	**2.834 (p = 0.007)**
**Unemployment Rate**	0.149	0.817 (p = 0.419)
**Median Household Income**	-0.148	-0.761 (p = 0.451)
**High School Graduation Rate**	-0.119	-0.812 (p = 0.422)
**Percent Uninsured**	-0.152	-0.981 (p = 0.333)
**Percent Specialist**	-0.252	-1.466 (p = 0.150)
**McKinsey top**	0.568	1.778 (p = 0.083)
**McKinsey middle**	-0.446	-1.951 (p = 0.059)
**McKinsey bottom**	-0.088	-0.322 (p = 0.749)

Supplementary Table 2. Coefficient estimates and p-values for Model 2 (r_k versus state-level political, economic and social measures). All continuous variables including r_k were centered and scaled prior to model fitting; therefore, their coefficients represent the expected number of standard deviations increase or decrease in r_k given a 1-standard deviation increase in the corresponding predictor variable. Values close to 1 or -1 indicate a strong positive or negative association with r_k. Coefficients for grouping variables (McKinsey top, middle and bottom) represent the expected value of r_k within each group, given mean values of all continuous variables. Results of a two-sided *t*-test on 40 degrees of freedom against the null hypothesis that each coefficient equals zero are also displayed.
