# Supplementary material for: Open data on industry payments to healthcare providers reveal potential hidden costs to the public
Source: Nat Commun. 2019 Sep 20;10:4314. doi: 10.1038/s41467-019-12317-z (PMC6754508; doi:10.1038/s41467-019-12317-z)
Supplement: Supplementary file 2 — Peer Review File [file 41467_2019_12317_MOESM2_ESM.pdf]

Reviewers' comments:

Reviewer #1 (Remarks to the Author):

Comments on “Open data on industry payments to healthcare providers reveals hidden costs to the public”

Summary: This is an interesting article analyzing the correlation between industry payments and Medicare costs. The authors have undertaken a difficult to accomplish analysis and have uncovered some interesting correlations between the two variables. I think the paper would be strengthened by some of my comments below.

Comments:

1. My main issue with this paper is that the authors, by their own admission, are looking at correlations between industry payments and medical costs and, in some instances, overstate the fact that these are correlations. Some examples include 1) the title (which implies that the manufacturer payments impact costs to the public) 2) the abstract (which references the “effect” of payments on healthcare costs), 3) describing a “relationship” between the two variables without cautioning that the relationship is a correlation, not a causation. It is certainly plausible that when pharmaceutical representatives and medical device manufactures are looking for providers to target with their payments, that they specifically target high cost providers (these are the ones where they may get the most bang for their buck). In my opinion, this reverse causality is certainly plausible and the authors need to address it thoroughly in order to couch their results. As for now, this is not even mentioned in the limitations.
2. As the authors note, one of the difficulties associated with analyzing the OP data in conjunction with another dataset is that the OP data lacks NPIs. The authors use the NPI public registry and find exact matches for 60% of providers in the OP dataset. This 60% sample is then used for all analyses. In order to enhance our interpretation of the results, it would be helpful to know a little bit more about the sample that is matched (60%) and not matched (40%). Is the 60% representative on characteristics of all physicians in the OP dataset? Are certain specialties/credentials over-represented and others under-represented? Are they representative in terms of actual payments; i.e. does the 60% sample represent roughly 60% of the dollars spent on these payments?
3. Figure 1 is very visually appealing but a little difficult to understand. Are these predicted results from the log-log regression model? Or do they represent raw numbers? Additionally, the authors report that there is some smoothing done for each line---what was the bandwidth chosen and how did they chose it?
4. The authors are primarily focusing on medical costs (non-drug), but do estimate additional models looking at drug costs and compare these estimates. I’m not quite sure how to interpret the comparison of these results---should we expect them to be more similar or less? Why? What is the mechanism by which drug payments should affect non-drug spending?
5. I do not understand the political results. They are interesting correlations, but the authors only control for a few demographics associated with the state and do not control for 1) the health differences across in the states and 2) the differences in the provider market across states. Both of these may influence the results you find. Particularly, some states have more specialists than others, and, if this is correlated with 2016 Trump popular vote, the results would be biased. While the authors are estimating correlations and do not claim causality, I believe their concluding statement “how a states’ political affiliation may shape the interface between medical providers and the healthcare industry” far overstates the implications of their results and goes too far on a limb.
6. The third paragraph in the conclusion overstates the results of this paper. The authors use “should” words about making consumers have more access to this data, but, their results do not support this. I think this paragraph needs to flag a bit more that these notes are speculative as they do not flow from the results of the study.

Reviewer #2 (Remarks to the Author):

This paper an interesting first effort to connect Sunshine data with overall HC costs. I have a few comments for how it could be strengthened:

1. The first Intro paragraph introduces the claim, which appears throughout the paper, that the U.S. is the only
"advanced" nation to allow financial ties between HC providers and manufacturers. This is not true - industry gifts and payments are common throughout most European countries, as well as Australia and China. If anything, ties are the rule, not the exception. Additionally, Sunshine laws exist in other countries, like France and Australia. This paragraph needs to be revised to reflect these conditions.

2. The first Intro paragraph also states that industry influence on providers "has not been clarified." This is inaccurate - there is an enormous literature on the impact of industry gifts and payments on providers' attitudes, knowledge, and behavior. The author(s) should acknowledge or summarize what is known, and what remains unknown, about this topic - and then contextualize their study within the literature.

3. At the top of page 3, there is a statement that OP data has "historically been examined by itself." First, I am not sure if the word 'historically' is appropriate given how recent OP is. Second, many studies have linked OP data to data on prescribing costs and generic vs. brand-name. These contributions are worthy of mention.

4. The recommendations for policymakers on page 7 seems only tangentially related to this study's research question, which concerns cost and not quality.

5. The recommendation to put patients "in charge of deciding how much they value the independence of their providers" needs more context. There is little to no evidence that patients are using OP data, or that they understand it. Research also shows that some patients believe industry ties indicate that provides are "important." Therefore it is not at all clear that a scorecard would cause consumers to select providers who are less conflicted and/or delivery lower-cost care. All these challenges and questions needs to be addressed.

6. The authors should be very careful not to attribute causation to correlation. Industry gifts and payments may indeed lead to costlier care, but they may also be "rewards" that companies give to high-prescribers. The author(s) should use caution when discussing findings and include a statement about this limitation in their discussion section.

In all, this paper presents interesting and novel research on an important topic; the above issues require substantial revision but would make the findings stronger and more compelling.

Response to Reviewers
NCOMMS-19-08787
Open data on industry payments to healthcare providers reveals *potential* hidden costs to the public

First, we wish to thank the reviewers for their positive feedback regarding the novelty and quality of our work.

- Reviewer 1 wrote: *This is an interesting article analyzing the correlation between industry payments and Medicare costs. The authors have undertaken a difficult to accomplish analysis and have uncovered some interesting correlations between the two variables.*
- Reviewer 2 wrote: *This paper an interesting first effort to connect Sunshine data with overall HC costs.*

We also appreciate the reviewers' constructive criticisms and suggestions for improving the paper. Based on the reviewers' comments, we have performed additional analyses, updated figures and revised the text to better communicate our findings. We believe the revised manuscript is much improved.

In short, both reviewers were supportive of our contribution, while raising a series of important issues. The reviewer comments and our corresponding responses and revisions center around three primary themes:

(A) **Avoiding the implication of causal claims.** We have now revised the title, abstract, introduction, results and discussion to eliminate any confusion regarding causal claims, which we do not attempt to make. We have also attempted to eliminate any language implying causality throughout the main text.

(B) **Expanding the works cited and positioning our contribution.** We have increased the literature reviewed to highlight that while previous work has demonstrated an association between industry ties and providers' drug choices, our work demonstrates an association between industry ties and providers' medical costs.

(C) **Adding robustness tests for our main models.** We have significantly improved our models and clarified our findings by including more controls and running additional specifications. Through these additional models, we find that (1) industry payments are associated with medical costs even after controlling for drug costs, and (2) a state's conservative advantage (but not the 2016 presidential election result) remains significantly and strongly related to the strength of the association between industry payments and medical costs after controlling for other state-level factors.

Below we present a point-by-point response to each of the points raised by the reviewers. Reviewer comments are shown in **bold face**, followed by our responses, with excerpts from the manuscript text displayed in blue.

Reviewer #1 (Remarks to the Author):

Comments:
1. My main issue with this paper is that the authors, by their own admission, are looking at correlations between industry payments and medical costs and, in some instances, overstate the fact that these are correlations. Some examples include

1) the title (which implies that the manufacturer payments impact costs to the public),
2) the abstract (which references the "effect" of payments on healthcare costs),
3) describing a "relationship" between the two variables without cautioning that the relationship is a correlation, not a causation.

Thank you for this feedback. Indeed, our study is "looking at correlations between industry payments and medical costs." To make this clearer and avoid any potential confusion for readers, we have revised the title (now, "Open data on industry payments to healthcare providers reveals *potential* hidden costs to the public") and the referred-to sentence in the abstract (now, "while it is known that these payments may pose conflicts of interest, *their relationship with overall* healthcare costs remains largely unknown."). On page 3, where we state the objective of the study, we now caution the reader that the relationship we report between industry payments and healthcare costs is not causal. We now state:

> While our goal was not to establish a direct causal link between industry payments and healthcare costs, we included several important controls to estimate this association robustly.

Additionally, throughout the text we have adjusted the language to reflect that we are looking at associations and not causal relationships. For example, when referring to our analysis and findings we have replaced the use of "effect" with "association", and of "increase" with "difference".

4) It is certainly plausible that when pharmaceutical representatives and medical device manufacturers are looking for providers to target with their payments, that they specifically target high cost providers (these are the ones where they may get the most bang for their buck). In my opinion, this reverse causality is certainly plausible and the authors need to address it thoroughly in order to couch their results. As for now, this is not even mentioned in the limitations.

Thank you for this important feedback. The conjecture that corporations may be in a sense making investments in high cost providers expecting a bigger "bang for their buck" is very intriguing and certainly plausible. We believe this is an important topic to address, which we neglected in our initial submission. We now attempt to address it thoroughly through four steps, which we now list briefly before going into more detail. First, (i) we now address the

non-causal nature of our analysis as a limitation in the discussion section and call for future work to further investigate the causal mechanisms behind our observed association. Second, (ii) we refer to the existing research looking at relationships between the medical device industry and physicians, which asserts that establishing causality is not a necessary objective. Third, (iii) we explain why we believe that reverse causality (costs → payments), which we agree is a plausible explanation of our findings, may be as much of a problem as direct causality (payments → costs). Finally, (iv) we extend our main model with medical costs as the dependent variable by additionally controlling for drug costs, which lends robustness to our previous findings and may partially allay concerns regarding the causal mechanisms behind those findings.

(i) In the discussion, we have revised the text to further communicate that our goal and models do not attempt to establish causality, and that this is a limitation of our analyses. We have expanded the paragraph on limitations on page 8 with the text shown below after the next point.

(ii) On 2017, the *Journal of the American Medical Association (JAMA)* produced an issue with a focus on conflicts of interest and with several articles devoted to interactions with industry (Vol. 317 issue 17). The articles in this issue and other recent work provide a number of helpful and relevant perspectives to our contribution. First, a viewpoint titled "Public Disclosure of Payments to Physicians From Industry"[1] reviewing existing analyses looking at the relationship between industry payments and prescribing behavior pointed out that those analyses had only established associations, and not causal relationships. This illustrates the current state of the literature looking at the relationship between industry payments and healthcare costs, which our paper builds upon. While establishing causality remains a worthy goal and may be ultimately more effective in provoking policy changes, to date this has proved elusive given the nature of the data.

In the discussion paragraph on limitations on page 8 we now state (changes in italics),

> Finally, our analysis is subject to the limitations of observational studies, *namely that it does not establish a causal effect of industry payments on healthcare costs. Given the nature of the data, previous studies investigating the relationship between industry ties and prescribing behavior have also been limited to correlational analyses.*[32] Future research is needed to determine the causal mechanisms relating industry contributions to costs.

Second, another viewpoint titled "Physicians, Industry Payments for Food and Beverages, and Drug Prescribing"[2] asserted that focusing on causality may not actually be necessary, as the financial relationships between physicians and the industry resulting from payments is problematic in and of itself. They state, "Focusing on such limitations [regarding causality], however, begs a broader question: is there any need to prove a causal relationship between industry payments to physicians and the prescribing of brand-name medications? First, industry-sponsored meals and other outright gifts may

be legal, but are there any reasons for physicians to either expect or accept them? [...] The American Medical Association's Code of Medical Ethics is clear that '[g]ifts to physicians from industry create conditions that carry the risk of subtly biasing—or being perceived to bias—professional judgment in the care of patients.'" While our paper does not adopt a position on whether industry payments are biasing providers, we do hope our study kickstarts a discussion on the broader impacts from industry payments on healthcare costs in the U.S. and leads to more work in this area. We have added a new discussion paragraph on causality, at the end of the discussion, on page 9:

> While establishing causality remains a worthy goal and may be ultimately more effective in provoking changes to policy, public opinion and provider behavior, to date this has proven elusive. Yet some have contended that establishing causality may not be a necessary goal. Steinbrook argued that the financial *relationships* between physicians and the industry resulting from payments are problematic in and of themselves, stating that "The American Medical Association's Code of Medical Ethics is clear that '[g]ifts to physicians from industry create conditions that carry the risk of subtly biasing—or being perceived to bias—professional judgment in the care of patients.'"[22]

(iii) Third, we believe that reverse causality, if at play, may be as dangerous as direct causality. That is, if physicians' levels of drug and medical costs are not being *caused* by industry payments, then an alternative explanation of the observed association is that they are being *rewarded* by them for being "big spenders". Such rewards would provide an incentive to physicians to increase spending with an expectation of future payments. Since both healthcare spending and industry payments are ongoing and frequently occurring events, there may indeed be a feedback loop whereupon spending begets payments, which in turn begets spending, and so on. While this would be difficult to disentangle econometrically due to the continuous and ongoing nature of both healthcare spending and industry payments, it is a plausible result of the reverse causality explanation. Further, if medical device companies are making investments in certain physicians (i.e., the "big spenders") expecting a return on their investment, this in fact implies that on average, those corporations expect that greater spending by those physicians will occur as a result. Such reverse causality (spending → payments) therefore may actually signal that medical device companies expect payments to result in greater spending (which they may be able to observe through their own transactional data).

Following the discussion paragraph on limitations, we have added a new paragraph on reverse causality on pages 8-9:

> In the context of our analysis, one possible alternative explanation of the observed association between total industry payments and total healthcare costs is that of reverse causality. That is, providers with high levels of spending may be targeted by the medical device industry as most likely to result in a return on investment. Yet this suggests that these corporations expect that on average, providers receiving payments will in turn increase

spending in ways that will benefit those corporations. Disentangling such a causal feedback loop would require more granular and detailed data on payments and healthcare expenditures than is currently available to the public.

(iv) Our main log-log regression model (model 1) used, for a given physician, the total annual medical costs as the response variable, with total industry payments, state and number of beneficiaries as predictors. The coefficient for total industry payments quantifies the association between payments and medical costs for physicians in a given state and with a given size of practice. We now include a second log-log regression model, where we also include total drug costs as a predictor. The coefficient for total industry payments in this model now tells us about the association between payments and medical costs for physicians in a given state, with a given size of practice, *and with a given level of drug costs.* We find that the estimated association between payments and medical costs remains substantial and statistically significant (0.091 versus 0.132 in the original model). While this does not disprove reverse causality (which we agree may be at least partly responsible for our findings), it does illustrate that even among providers with comparable drug costs (which we might expect pharmaceutical companies to observe and therefore respond to through payments), variability in payments is related to variability in medical costs. Further, this model serves to extend our contributions to the existing literature, which has, to the best of our knowledge, thus far only investigated relationships between drug costs and industry payments.

The additional robustness check is included in the section, **"The association between industry payments and medical costs"** in a sub-section titled **"This new association with medical costs goes beyond the previously suggested association with drug costs"** on page 4. See also response to point (4) below.

2. As the authors note, one of the difficulties associated with analyzing the OP data in conjunction with another dataset is that the OP data lacks NPIs. The authors use the NPI public registry and find exact matches for 60% of providers in the OP dataset. This 60% sample is then used for all analyses. In order to enhance our interpretation of the results, it would be helpful to know a little bit more about the sample that is matched (60%) and not matched (40%). Is the 60% representative on characteristics of all physicians in the OP dataset? Are certain specialties/credentials over-represented and others under-represented? Are they representative in terms of actual payments; i.e. does the 60% sample represent roughly 60% of the dollars spent on these payments?

We apologize for omitting this information in the original submission. First, we must note a correction to our original report. In further analyzing the matched subset, we realized that in fact 90.4% of the OP providers receiving any payments in 2016 (the year we analyzed) were successfully matched. Our previous report that 60% of providers were matched was based on the full set of providers appearing in the OP dataset's Physician Supplementary File, which contains information on a much larger number of providers, including but not limited to those

receiving payments in 2016. As the percentage of matched providers is much higher than we previously reported, we hope that concerns about the representativeness of the matched sample are somewhat allayed. In any case, below we summarize some key characteristics of the matched and unmatched samples based on the 2016 OP data.

First, in terms of the amount and number of payments, the total payments in the matched sample is $1.95B, which represents 91.8% of the overall total of $2.12B. The mean and median amount of payments for providers in the matched and unmatched sample are shown in the table below, along with the mean and median *number* of payments for providers in both groups. The total and number of payments for the matched sample is slightly higher than that of the unmatched sample, but overall the two groups are fairly comparable in terms of payments.

Result	Mean (median) total industry payments	Mean (median) number industry payments
Matched	$2,736 ($124)	15.2 (3)
Unmatched	$2,269 ($118)	12.6 (3)

In terms of physician primary type, the distribution within each group (matched and unmatched) is shown below. The two groups are also fairly comparable here, although the unmatched group has a larger representation of medical doctors and fewer dentists.

Physician primary type	Proportion of matched	Proportion of unmatched
Chiropractor	0.003	0.002
Doctor of Dentistry	0.125	0.065
Doctor of Optometry	0.044	0.040
Doctor of Osteopathy	0.073	0.080
Doctor of Podiatric Medicine	0.017	0.022
Medical Doctor	0.738	0.791
**Total**	**1.000**	**1.000**

To compare the distribution of specialties, since there are over 400 different types of specialties in the OP data, in the table below we show the top 10 most frequent specialities within the matched group. The numbers represent the proportion of each group represented by a

particular specialty. We do not see any major differences between the two groups in terms of the distribution of specialties.

Physician specialty (only showing top 10 specialties sorted by percent matched)	Proportion of matched	Proportion of unmatched
Allopathic & Osteopathic Physicians|Internal Medicine	0.113	0.129
Allopathic & Osteopathic Physicians|Family Medicine	0.096	0.093
Dental Providers|Dentist|General Practice	0.055	0.026
Eye and Vision Services Providers|Optometrist	0.042	0.038
Allopathic & Osteopathic Physicians|Pediatrics	0.039	0.031
Dental Providers|Dentist	0.039	0.021
Allopathic & Osteopathic Physicians|Obstetrics & Gynecology	0.033	0.031
Allopathic & Osteopathic Physicians|Internal Medicine|Cardiovascular Disease	0.03	0.03
Allopathic & Osteopathic Physicians|Anesthesiology	0.026	0.023
Allopathic & Osteopathic Physicians|Surgery	0.025	0.029
**More rows omitted for brevity**	...	...

In the Methods section, in the description of Dataset 3 on pages 13-14, we now state:

> Of the 636,871 unique providers appearing in the 2016 OP dataset, we found exact matches for 576,144 (90.4%), whose collective payments represented 91.8% of the total 2016 general payments. The matched and unmatched providers did not differ substantially in terms of the amount or number of payments, or in the types of providers represented. Of the matched providers, 374,766 also appeared in the MPOS 2016 dataset.

3. Figure 1 is very visually appealing but a little difficult to understand. Are these predicted results from the log-log regression model? Or do they represent raw numbers? Additionally, the authors report that there is some smoothing done for each line---what was the bandwidth chosen and how did they chose it?

Thank you for pointing out the potential confusion around this figure. These plots are not based on the log-log regression model, but rather are exploratory plots that helped to guide our regression model framework. To aid the reader, we have changed the caption title to,

"***Exploring the relationship* between industry payments to providers and medical costs**". Regarding the bandwidth, we used a span of 0.75 (meaning that 75% of all the observations were used in each local polynomial regression model), which is the default setting in the R loess function. We did not attempt to adjust the settings of the loess function to better illustrate the observed relationship. We have added more details on the loess plots to the Methods section on page 14 (changes in italics):

> To visually investigate the relationship between industry payments and medical costs, given the large number of observations in our dataset we performed loess smoothing of the scatterplot of log payments versus log medical costs (both total and per beneficiary) within each state (**Figure 1**). *The curves for each state were obtained using stat_smooth of the ggplot2 R package (https://ggplot2.tidyverse.org/) using the default parameter values and methods of the R loess function, including a span of 0.75 (meaning that 75% of all the observations were used in each model) and degree of 2 for the local polynomial regression models.*

We now direct the reader to these details in the first sentence of the figure caption, which reads "Each line represents a loess smoother of the scatterplot of the providers within each state *(see* ***Methods*** *for details)*."

4. The authors are primarily focusing on medical costs (non-drug), but do estimate additional models looking at drug costs and compare these estimates. I'm not quite sure how to interpret the comparison of these results---should we expect them to be more similar or less? Why? What is the mechanism by which drug payments should affect non-drug spending?

Thank you for this comment. In the revision, we further examined and contextualized the two different sources of costs. Following the presentation of the main model focused on medical costs, we added a new sub-section on page 4 titled, **"This new association with medical costs goes beyond the previously suggested association with drug costs."** The text of the new section appears after our response below.

To summarize our previous findings, we had reported an association between industry payments and medical costs, as well as between industry payments and drug costs. The latter finding is somewhat expected, given the previous findings in the literature regarding the association between industry payments and prescribing behavior of physicians. However, the medical costs effect is novel, as to the best of our knowledge all previous studies have examined only drug costs. In the original submission, we reported both associations in order to put the medical costs effect in context, as the reader can compare it to the more expected drug costs effect. (The two effects can be directly compared, since the log-log regression slopes are interpreted as the percent difference in costs associated with a percent difference in payments across providers.) Specifically, we find that a 10% difference in industry payments is associated with a 1.3% difference in medical costs (Model 1) and a 1.8% difference in drug costs (Model

1a). The magnitude of the association with medical costs was frankly somewhat surprising to us, since the association with drug costs has been the major focus of previous investigations.

However, in the original submission we failed to highlight the fact that medical costs are much higher than drug costs for each provider. Specifically, for providers within the middle range of industry payments (the subset used in the log-log model, see Figure 1), the median annual medical costs are $90,646[1] per provider, while the median annual drug costs (among prescribing providers, i.e. those with non-zero drug costs) are only $5,862 per physician. Therefore, the potential impact of increased medical costs in dollars to the U.S. healthcare system may be profound. For example, for a "typical" provider (one with median levels of medical and drug spending), a 1.3% change in medical costs would amount to over $1,100[1], while 1.8% change in drug costs would only represent approximately $100. Though our analysis does not claim that industry spending is *causing* such changes, this contrast highlights that medical costs will be important to understand and control in order to reduce or maintain overall healthcare spending in the U.S.

To more fully understand the association between medical costs and industry payments and to establish the degree of independence between medical and drug costs, in the revised manuscript we fit an additional, more comprehensive model to the data (Model 1b). First of all, we know that medical and drug costs are somewhat correlated (Spearman rank correlation = 0.41 among providers in middle range of industry payments). So, we now include a robustness check to ensure that the observed association between industry payments and medical costs is not simply due to a spurious correlation. Specifically, we fit a model with medical costs (log scale) as the dependent variable, yet including drug costs (log scale) as an independent variable, in addition to industry payments (log scale), state and number of beneficiaries (log scale). In this model, the coefficient for industry payments represents the percentage difference in medical costs associated with a 1% change in industry payments, holding the provider state, number of beneficiaries, *and annual drug costs* fixed. Therefore, this coefficient represents the association of industry payments with medical costs ***above and beyond*** any association with drug costs. In this model, we find a 0.9% difference in medical costs associated with a 10% difference in industry payments (compared with 1.3% in our original model not controlling for drug costs), which remains substantial and statistically significant.

We would like to point out that, we consider the mechanisms behind the association between industry payments and medical costs beyond the scope of this paper and the data sources used. Yet, it is important to note that the industry payments are primarily coming from medical

[1] Note that the median annual medical cost value of $90,646 and the associated "effect size" of approximately $1,100 are different from the original submission, where the median annual medical cost value was $41,700 with an associated effect size of $540. These original values were based on the full sample of providers, rather than the "middle" subset (those with industry payments between the 1st and 3rd quartiles) used in the regression model. Since we cannot extrapolate the regression coefficients outside of this range, where the relationship deviates from linearity, it is more appropriate to utilize the middle subset to quantify the levels of drug and medical spending of a "typical" provider.

device corporations, which include but are not limited to pharmaceutical companies. Therefore, the industry's attempts to influence medical providers may extend beyond the prescription of name brand versus generic drugs to patients. We believe that this association between industry payments and medical costs, which we observe both concurrently and in addition to the association between industry payments and drug costs previously reported in the literature, represents a hidden but substantial cost to healthcare consumers and the Medicare system. Physician prescribing behavior may, in fact, be just the tip of the iceberg.

The new sub-section on pages 4-5 reads:

> **This new association with medical costs goes beyond the previously suggested association with drug costs.** The association reported above is distinct from that between industry payments and prescribing behavior that has been previously reported.[10] Consistent with these previous reports, we observed an association between industry payments and drug costs, with a 10% difference in payments being associated with an estimated 1.8% higher drug costs (**Model 1b;** see **Methods** for details). To further test the robustness of the new association with *medical* costs, we re-estimated the association controlling for the provider's total drug costs (**Model 1c**; see **Methods** for details). In this model, the estimate of β_1 was 0.091 (95% CI: [0.086, 0.095]), so that a 10% difference in industry payments was associated with a 0.91% difference in medical costs, given fixed drug costs. This test served three primary purposes. First, it established that the association between industry payments and medical costs was *not* spurious or due to the correlation between providers' medical and drug costs (Spearman rank correlation = 0.43 among providers with non-zero drug costs). Second, it showed a significant association between industry payments and medical costs *above and beyond* the association between such payments and drug costs suggested by previous work. Finally, it provided an additional control to establish an association between industry payments and medical costs among physicians that are comparable in terms of several key factors (namely location, size and drug costs). We note that this robustness test complements, rather than replaces, the original model having a 1.3% effect size, as drug and medical costs tend to increase together in practice.[9]

In addition, the subsequent subsection on page 5 now reads (changes in italics):

> ***Putting the observed association between industry payments and medical costs in context.*** To put a 1.3% *difference* in total medical costs in context, consider a "typical" provider *within the middle range of industry payments,* with industry payments equal to the median of $250 and total Medicare medical costs equal to the median of *$90,646*. A 10% difference of $25 in industry payments would then be associated with an expected difference of approximately *$1,100* in total medical costs. *Controlling for drug costs, the expected difference in medical costs would be 0.91%, or $825.* Such differences in medical costs are much larger than the differences in industry payments with which they are associated. Therefore, even a small numerical difference in payments is associated with a much larger difference in expected medical costs. *Further, although the estimated effect size of 1.8% for drug costs is larger than the 1.3% estimated effect size for medical costs, physicians tend to have much higher medical costs than drug costs. In fact, 61% of the providers in this analysis had no drug costs. Among providers within the middle range of industry payments, the median non-zero drug costs were $5,862, so that a 10% difference in industry payments would be associated with a difference in drug costs of 1.8% or approximately $100.*

5. I do not understand the political results. They are interesting correlations, but the authors only control for a few demographics associated with the state and do not control for 1) the health differences across in the states and 2) the differences in the provider market across states. Both of these may influence the results you find. Particularly, some states have more specialists than others, and, if this is correlated with 2016 Trump popular vote, the results would be biased. While the authors are estimating correlations and do not claim causality, I believe their concluding statement "how a states' political affiliation may shape the interface between medical providers and the healthcare industry" far overstates the implications of their results and goes too far on a limb.

Thank you for these comments and recommendations. First, we have revised our concluding statement on page 7 to read, "how a state's political affiliation may *be related to* the interface between medical providers and the healthcare industry." We hope this allays any concerns about the implication of causality. Second, following these suggestions, we have revised the state-level model to control for differences in the provider market and health differences across states. As a result, the overall model fit is improved (new adjusted R-squared of 0.4167 versus original submission adjusted R-squared of 0.3909) and we believe that the findings are now clearer. Below, we explain our approach to controlling for these variables in detail. To first summarize, none of the demographic predictor variables were found to be statistically significant (Figure 5). Only the coefficient for conservative advantage was found to be significative (Figure 5, indicated in red). Importantly, this is consistent in terms of both effect size and statistical significance with the results reported in our first submission.

However, incorporating the new variables does show a reduced contribution of the 2016 Trump popular vote, due to strong correlations between the election results and health differences across states. We appreciate the advice to examine the robustness of our findings by controlling for additional important state-level factors. As a result of the suggested analyses, we have excluded Model 2 (with election results as the DV) from the revised manuscript. We believe that Conservative Advantage is the most important measure at hand, since it represents a long-term measure of a state's political leaning. This is more likely to be robust to election-to-election state-wide variations and to be related to the industry-provider interface than a single presidential election.

Here we now provide more details about how we approached the new analyses and model fitting in response to the reviewer's suggestion. First of all, we collected a new data source McKinsey Co. Health Rankings (now described in the **Methods** in **Dataset 5**). This data set is a reliable source of information about healthcare quality in a state. We used this new data set in combination with the previous data sources to sort out the influence of additional demographic variables. In Table R1 below, we list several key metrics from the original model (Model 2orig in Table R1), a new model controlling for provider market differences through a new variable representing the percentage of specialists (Model 2S in Table R1), and several candidate models to additionally control for health differences (Models 2S-A, 2S-B and 2S-C in Table R1). To measure health differences across states, we considered McKinsey Health Rankings,

grouped into thirds (Model 2S-A), America's Health Rankings, grouped into thirds (Model 2S-B), and infant mortality and colorectal cancer screening rates (Model 2S-C). Infant mortality and colorectal cancer screening rates were selected among several candidate continuous variables typically used as measures of population health (e.g., adult obesity rates, heart disease death rates, influenza vaccination coverage rates), as they contributed the most predictive power to the model and were not collinear.

Model 2S-A is the strongest in terms of adjusted R-squared and the joint significance of the health variables as assessed with an F-test. We therefore adopt it for our state-level model in the revised manuscript. Note that none of the new variables are statistically significant, either individually or jointly, based on an F-test versus the original model (p=0.2192). Yet the overall model fit is improved, and we believe that these new variables represent important state-level controls. We have now updated the text of section, **"Geographic variance relates to political and population factors"** on pages 6-7, Figure 3 on page 7, and Table S2 of the Supplementary Information to reflect these changes.

Interestingly, variables such as the overall health of a state, health insurance coverage rates and percentage of specialists, are not statistically significant in our model. While these variables would likely be associated with *medical costs per se*, they do not appear to be related to the *strength of the association between industry payments and those costs*. This highlights a potential source of confusion for readers, as our model is essentially looking at the correlation of a correlation. This can be challenging to communicate, so we have sought to be more explicit about this model's objectives in the revised manuscript. Before reporting the model results, we now state on page 6 (italics appearing in text),

> Note that this model is not concerned with the *amount* of medical costs or industry payments, but rather the *strength of the association* between providers' industry payments and medical costs (i.e., r_k) after controlling for other key provider-level factors.

Model	Model description	Adj. R^2	Conservative Advantage: Estimate	Conservative Advantage: p-value	F-test vs Model 2: p-value	Comments
Model 2orig	Model 2 from original submission	0.3909	0.646	0.0011	NA	
Model 2S	Model 2orig + percentage specialists	0.3787	0.649	0.0012	NA	New var not significant (p=0.713)
**Model 2S-A**	**Model 2S + McKinsey health rankings (adopted in revision)**	**0.4176**	**0.706**	**0.0013**	**0.136**	**None of the grouping variables is significant and the Anova is not significant**

Model 2S-B	Model 2S + America's Health Rankings	0.3909	0.551	0.0143	0.292	None of the grouping variables is significant and the Anova is not significant
Model 2S-C	Model 2S + infant mortality and colorectal cancer screening	0.4053	0.597	0.0075	0.153	Neither infant mortality nor cancer screening is significant separately or together

Table R1. Comparison of models looking at the association between state-level factors and the strength of the association between industry payments and medical costs in a state. Model 2orig is the version of Model 2 from the original submission; Model 2S controls for the percentage of specialists in the provider market; and Models 2S-A, 2S-B and 2S-C additionally control for the healthcare quality in each state. **Model 2S-A has the best fit as assessed with adjusted R-squared and is therefore adopted as Model 2 in the revised manuscript.** An F-test comparing Model 2S-A to Model 2orig shows that jointly, the new variables are not statistically significant (p=0.2192), nor are they significant when considered individually. Yet they represent important controls and improve overall model fit as assessed with adjusted R-squared (0.4176 in Model 2S-A versus 0.3909 in Model 2orig).

6. The third paragraph in the conclusion overstates the results of this paper. The authors use "should" words about making consumers have more access to this data, but, their results do not support this. I think this paragraph needs to flag a bit more that these notes are speculative as they do not flow from the results of the study.

Thank you for pointing this out. We have rewritten the paragraph in question to (1) better summarize what the previous literature has suggested as next steps for OP and (2) provide our own ideas for what might allow patients to digest and utilize the information available in OP. Ultimately, we believe that the debate in the academic literature, including our own work here, has been dominated by trying to understand the influence of the healthcare industry on medical providers. While this is a worthy goal, we believe a more patient-centric approach is necessary from future work. At the core, we aim to provide the reader with a question: what should patients make of all this information, including the findings of our paper? Our suggestion, which is rooted in previous work examining the role of transparency in other contexts, is aimed at providing and synthesizing OP data at the point of service. We hope that we are better able to convey our ideas with the revised paragraph on page 8:

> To make actionable use of the transparency provided by the OP system, we believe more work is needed to help patients access and interpret the data. As a 2017 JAMA article argued, "Transparency regarding public disclosure of payments to physicians from industry, and the relationship between payments and prescribing is still in its infancy. Going forward, as additional data become available, it will be imperative to continue improving the tools available for consumers and provide information and context on how to use them."[32] Several possible strategies for improved use of OP data to empower healthcare consumers have been suggested, including flagging unusually high payments,[21] going beyond disclosure to incentives,[41] educating patients on how to interpret OP data,[42] and expanding the reach of the OP system.[43] A suggestion we would like to put forth is to

provide OP information to the public at the point of service, a strategy that has been utilized effectively in other contexts, including restaurant hygiene, auto safety ratings and appliance energy efficiency metrics.[39] For example, restaurant hygiene scorecards have led to important decreases in foodborne illness.[44] By giving the public digestible information on physicians' industry ties at the point of service, patients would be put in charge of deciding how much they value the independence of their providers from the healthcare industry. An important first step, however, is to investigate how consumers can accurately gauge and interpret the information in the OP system and how they may act in response.

Reviewer #2 (Remarks to the Author):

This paper an interesting first effort to connect Sunshine data with overall HC costs. I have a few comments for how it could be strengthened:

1. The first Intro paragraph introduces the claim, which appears throughout the paper, that the U.S. is the only "advanced" nation to allow financial ties between HC providers and manufacturers. This is not true - industry gifts and payments are common throughout most European countries, as well as Australia and China. If anything, ties are the rule, not the exception. Additionally, Sunshine laws exist in other countries, like France and Australia. This paragraph needs to be revised to reflect these conditions.

Thank you pointing this out. Following your suggestion, we deleted these comments, which were not central to our message. That is, our study is only concerned with U.S. data on medical providers' industry ties and healthcare costs. We hope to avoid any misleading statements and be more targeted in the revised manuscript.

2. The first Intro paragraph also states that industry influence on providers "has not been clarified." This is inaccurate - there is an enormous literature on the impact of industry gifts and payments on providers' attitudes, knowledge, and behavior. The author(s) should acknowledge or summarize what is known, and what remains unknown, about this topic - and then contextualize their study within the literature.

Thank you for your suggestion. We believe we should have been more specific in our phrasing and appreciate the opportunity to clarify our message. What we wanted to communicate is that the relationship between the healthcare industry and medical providers continues to be a topic of interest for academics and the public. We did not wish to imply there has not been in any work in this area. We have changed the sentence in question (on page 2) to: "Concern has been put forth about the extent and manner of the industry's influence on the behavior of individual healthcare providers and the implications for healthcare costs.[10] As of today, the full extent of such influence is not well understood."

More importantly, following your suggestion, we now review the related literature and identify the specific gap our work aims to fill. For example, we now cite the 2017 JAMA editorial by DeJong and Dudley introducing an issue with several articles devoted to the relationship between the healthcare industry and medical providers[12]. The central article of that issue was a study associating industry payments with medical provider drug prescribing behavior, Larkin et al. (2017)[13]. These and other studies suggest there is a relationship between industry payments and *prescription choices.* Our paper builds on this work but asks a different question: how are industry payments associated with *medical* costs in the U.S. healthcare system?

In the original submission we referred to some of this work (namely, “Existing studies in this area have demonstrated a relationship between payments from specific pharmaceutical companies (i.e., opioid manufacturers) and specific prescribing behavior (i.e., opioid prescriptions) of medical providers [17,23].”), but we were remiss to not reference more recent work in this area. We now attempt to provide more context through new language appearing in the introduction on pages 2-3:

> Substantial research has focused on the impact of industry payments and more generally medical marketing on providers' attitudes and behavior in the U.S.[24] For example, higher payments to medical providers by the healthcare industry have been linked to the prescription of brand-name drugs, even when equally effective generic substitutes are available.[25–30] In a more pernicious context, previous work demonstrated a relationship between payments to medical providers from opioid manufacturers and an increase of opioid prescriptions, which may have contributed to the ongoing opioid epidemic.[18,31] These findings have also been documented in the press by Propublica, a nonprofit investigative news organization, in their “Dollars for Docs” report.[32] While most of these studies have focused on the healthcare industry’s influence over drug prescriptions,[10] medical spending on services (e.g., procedures, utilization) represents a much larger fraction of overall healthcare costs than drug spending. For example, in the 2016 Medicare data we report on below, allowed medical costs amounted to over $82B, or over five times the total drug costs of $15B.

We hope the revised manuscript more accurately and thoroughly represents the previous work that has been done in this area and places our contribution into that context.

3. At the top of page 3, there is a statement that OP data has "historically been examined by itself." First, I am not sure if the word 'historically' is appropriate given how recent OP is. Second, many studies have linked OP data to data on prescribing costs and generic vs. brand-name. These contributions are worthy of mention.

Thank you for pointing this out. We agree that given the recency of OP data, the word “historically” may not be the best choice. As such, we have rewritten that sentence to better communicate our point, which is that there is an inherent difficulty in linking OP data because the National Provider Identifier (NPI) is not included. We hope that the revised language, which appears on page 3, better represents our point:

> The paucity of research on the association between industry payments and overall healthcare costs may be in part due to the challenge in linking OP to healthcare data on individual providers, because the National Provider Identifier (NPI) is missing from the OP dataset.[22,24] To address this difficulty, we programmatically matched over 90% of the NPIs in the 2016 OP dataset. We used the NPI-matched OP data for all the analyses in this paper, which we make openly available for the research community to facilitate future research.

4. The recommendations for policymakers on page 7 seems only tangentially related to this study's research question, which concerns cost and not quality.

Thank you for this comment. Our recommendations for policymakers do not focus on quality – we apologize if we inadvertently suggested that. To ensure that we are not sending the wrong message, we have removed all references to quality in the discussion.

Our recommendations center around two themes: (a) going beyond transparency to provide patients easier access to OP data and (b) including the National Provider Identifier (NPI) in the OP system. Comment (5) and our response below address (a), so here we will focus on (b). The goal of including the NPI in OP would be to allow other researchers to better utilize the OP data to understand the implications of industry contributions on many facets of the healthcare system, including costs, quality, etc. Without this important linking variable, such research is more difficult. For example, in the dataset we compiled for this paper, we had to programmatically search for the NPI of each provider in OP by querying NPI public registry. Though our matching was successful 90.4% of the time, the lack of a complete linkage may introduce selection bias. More importantly, performing such matching is time-consuming and requires technical expertise that may hinder many researchers. If the NPI were to be included in the OP system, researchers could easily link OP data with other CMS datasets that include information on both cost and quality of medical providers and organizations. We hope this clarifies our intent in providing this recommendation for policymakers. The revised discussion paragraph on this recommendation appears on page 8 and reads (changes in italics):

> We hope our study will spur future research on the implications of industry payments in the healthcare system, with the ultimate goal of impacting policy making. Such research is currently limited by the exclusion of the NPI from the OP system. Inclusion of the NPI in OP would allow researchers to link data on industry payments directly to the other datasets provided by CMS. *While we were able to match 90.4% of the providers in the 2016 OP data programmatically, the lack of a complete linkage introduces the possibility of selection bias. Such linkage is also time-consuming and requires substantial effort and expertise. This is a fundamental limitation of the OP system in its current state. Interestingly, this problem was foreseen and reported on prior to the rollout of OP in 2011.*[45]

5. The recommendation to put patients "in charge of deciding how much they value the independence of their providers" needs more context. There is little to no evidence that patients are using OP data, or that they understand it. Research also shows that some patients believe industry ties indicate that provides are "important." Therefore it is not at all clear that a scorecard would cause consumers to select providers who are less conflicted and/or delivery lower-cost care. All these challenges and questions needs to be addressed.

Thank you for these comments and insights. We agree that there is little evidence that patients are aware of, are using, or understand OP data. A national survey of over 3,000 adults concluded, "very few Americans know whether their own doctor has received industry payments or are aware that payment information is publicly available."[23] We also agree that the topic of how patients perceive medical providers' industry ties deserves more attention. One survey of 1,701 U.S. registered voters found that 74% of respondents believed industry payments

influence how a provider will treat a patient, and 51% of respondents would be less likely to choose a provider who has received industry payments.[24] Conversely, Rose et al. (2019) found in a randomized trial that disclosing industry payments did not affect patients' trust in their provider or lead to missed appointments.[25] Yet other recent work has shown that patient trust is diminished after patients are exposed to Open Payments information.[26]

We believe that more work is needed to understand how patients can best understand OP data and how they respond to different forms of communication and education surrounding industry ties. In our recommendation, we simply want to suggest that putting patients in charge at the point of service, instead of making information available only through an online database, could be one way to empower consumers. That being said, this recommendation is speculative, and we have attempted to make this clear in the revised manuscript. We have also aimed to put our recommendations in the context of previous work that has sought to provide recommendations for improved use of OP data. The updated discussion paragraph on this topic appears on page 8 and reads:

> To make actionable use of the transparency provided by the OP system, we believe more work is needed to help patients access and interpret the data. As a 2017 JAMA article argued, "Transparency regarding public disclosure of payments to physicians from industry, and the relationship between payments and prescribing is still in its infancy. Going forward, as additional data become available, it will be imperative to continue improving the tools available for consumers and provide information and context on how to use them."[32] Several possible strategies for improved use of OP data to empower healthcare consumers have been suggested, including flagging unusually high payments,[21] going beyond disclosure to incentives,[41] educating patients on how to interpret OP data,[42] and expanding the reach of the OP system.[43] A suggestion we would like to put forth is to provide OP information to the public at the point of service, a strategy that has been utilized effectively in other contexts, including restaurant hygiene, auto safety ratings and appliance energy efficiency metrics.[39] For example, restaurant hygiene scorecards have led to important decreases in foodborne illness.[44] By giving the public digestible information on physicians' industry ties at the point of service, patients would be put in charge of deciding how much they value the independence of their providers from the healthcare industry. An important first step, however, is to investigate how consumers can accurately gauge and interpret the information in the OP system and how they may act in response.

6. The authors should be very careful not to attribute causation to correlation. Industry gifts and payments may indeed lead to costlier care, but they may also be "rewards" that companies give to high-prescribers. The author(s) should use caution when discussing findings and include a statement about this limitation in their discussion section.

Thank you for this important and insightful feedback. Indeed, our study is looking at correlations and does not establish causal ties. To make this clearer and avoid any potential confusion for readers, we have revised the title (now, **"Open data on industry payments to healthcare providers reveals *potential* hidden costs to the public"**) and have revised the abstract and main text throughout to avoid causal implications, avoiding the use of words

like “relationship”, “effect” and “increase” whenever possible. On page 3, where we state the objective of the study, we now caution the reader that the relationship we report between industry payments and healthcare costs is not causal:

> While our goal was not to establish a direct causal link between industry payments and healthcare costs, we included several important controls to estimate this association robustly.

Regarding reverse causality, the conjecture that corporations may be rewarding high-prescribers is very intriguing and certainly plausible. We believe this is an important topic to address, which we neglected in our initial submission. We now attempt to address it thoroughly through four steps, which we now list briefly before going into more detail. First, (i) we now address the non-causal nature of our analysis as a limitation in the discussion section and call for future work to further investigate the causal mechanisms behind our observed association. Second, (ii) we refer to the existing research looking at relationships between the medical device industry and physicians, which asserts that establishing causality is not a necessary objective. Third, (iii) we explain why we believe that reverse causality (costs → payments), which we agree is a plausible explanation of our findings, may be as much of a problem as direct causality (payments → costs). Finally, (iv) we extend our main model with medical costs as the dependent variable by additionally controlling for drug costs, which lends robustness to our previous findings and may partially allay concerns regarding the causal mechanisms behind those findings.

i) In the discussion, we have revised the text to further communicate that our goal and models do not attempt to establish causality, and that this is a limitation of our analyses. We have expanded the paragraph on limitations on page 8 with the text shown below after the next point.

ii) On December 2018, the *Journal of the American Medical Association (JAMA)* produced an issue with a focus on conflicts of interest and with several articles devoted to interactions with industry (Vol 320, No. 22). The articles in this issue and other recent work provide a number of helpful and relevant perspectives to our contribution. First, a viewpoint titled “Public Disclosure of Payments to Physicians From Industry”[1] reviewing existing analyses looking at the relationship between industry payments and prescribing behavior pointed out that those analyses had only established associations, and not causal relationships. This illustrates the current state of the literature looking at the relationship between industry payments and healthcare costs, which our paper builds upon. While establishing causality remains a worthy goal and may be ultimately more effective in provoking policy changes, to date this has proved elusive given the nature of the data on payments and healthcare costs.

In the discussion paragraph on limitations on page 8 we now state (changes in italics),

> Finally, our analysis is subject to the limitations of observational studies, *namely that it does not establish a causal effect of industry payments on healthcare costs. Given the nature of the data, previous studies investigating the relationship between industry ties and prescribing behavior have also been limited to correlational analyses.*[32] Future research is needed to determine the causal mechanisms relating industry contributions to costs.

Second, another viewpoint titled "Physicians, Industry Payments for Food and Beverages, and Drug Prescribing"[2] asserted that focusing on causality may not actually be necessary, as the financial relationships between physicians and the industry resulting from payments is problematic in and of itself, stating:

> Focusing on such limitations [regarding causality], however, begs a broader question: is there any need to prove a causal relationship between industry payments to physicians and the prescribing of brand-name medications? First, industry-sponsored meals and other outright gifts may be legal, but are there any reasons for physicians to either expect or accept them? [...] The American Medical Association's Code of Medical Ethics is clear that '[g]ifts to physicians from industry create conditions that carry the risk of subtly biasing—or being perceived to bias—professional judgment in the care of patients.'

While our paper does not adopt a position on whether industry payments are biasing providers, we do hope our study kickstarts a discussion on the broader impacts from industry payments on healthcare costs in the U.S. and leads to more work in this area. We have added a new paragraph on causality at the end of the discussion, on page 9:

> While establishing causality remains a worthy goal and may be ultimately more effective in provoking changes to policy, public opinion and provider behavior, to date this has proven elusive. Yet some have contended that establishing causality may not be a necessary goal. Steinbrook argued that the financial *relationships* between physicians and the industry resulting from payments are problematic in and of themselves, stating that "The American Medical Association's Code of Medical Ethics is clear that '[g]ifts to physicians from industry create conditions that carry the risk of subtly biasing—or being perceived to bias—professional judgment in the care of patients.'"[22]

(iii) Finally, we believe that reverse causality, if at play, may be as dangerous as direct causality. That is, if physicians' levels of drug and medical costs are not being *caused* by industry payments, then an alternative explanation of the observed association is that they are being *rewarded* by them for being "big spenders". Such rewards would provide an incentive to physicians to increase spending with an expectation of future payments. Since both healthcare spending and industry payments are ongoing and frequently occurring events, there may indeed be a feedback loop whereupon spending begets payments, which in turn begets spending, and so on. While this would be difficult to disentangle econometrically due to the continuous and ongoing nature of both healthcare spending and industry payments, it is a plausible result of the reverse causality explanation. Further, if medical device companies are making investments in certain physicians (i.e., the "big spenders") expecting a return on their investment, this in

fact implies that on average, those corporations expect that greater spending by those physicians will occur as a result. Such reverse causality (spending → payments) therefore may actually signal that medical device companies expect payments to result in greater spending (which they may be able to observe through their own transactional data).

Following the discussion paragraph on limitations, we have added a new paragraph on reverse causality on pages 8-9:

> In the context of our analysis, one possible alternative explanation of the observed association between total industry payments and total healthcare costs is that of reverse causality. That is, providers with high levels of spending may be targeted by the medical device industry as most likely to result in a return on investment. Yet this suggests that these corporations expect that on average, providers receiving payments will in turn increase spending in ways that will benefit those corporations. Disentangling such a causal feedback loop would require more granular and detailed data on payments and healthcare expenditures than is currently available to the public.

iv) Our main log-log regression model has, for a given physician, total annual medical costs as the response, with total industry payments, state and number of beneficiaries as predictors. The coefficient for total industry payments quantifies the association between payments and *medical costs* for physicians in a given state and with a given size of practice. We now include a second log-log regression model, where we also include total *drug costs* as a predictor. The coefficient for total industry payments in this model now tells us about the association between payments and medical costs for physicians in a given state, with a given size of practice, *and with a given level of drug costs.* We find that the estimated association between payments and medical costs remains substantial and statistically significant (0.091 versus 0.132 in the original model). While this does not disprove reverse causality (which we agree may be at least partly responsible for our findings), it does illustrate that even among providers with comparable drug costs (which we might expect pharmaceutical companies to observe and therefore respond to through payments), variability in payments is related to variability in medical costs. Further, this model serves to extend our contributions to the existing literature, which has, to the best of our knowledge, thus far only investigated relationships between drug costs and industry payments.

The additional robustness check is included in the section, **"The association between industry payments and medical costs"** in a sub-section titled **"This new association with medical costs goes beyond the previously suggested association with drug costs"** on pages 4-5.

References

1. Ornstein, C. Public Disclosure of Payments to Physicians From Industry. *JAMA* **317**, 1749–1750 (2017).
2. Steinbrook, R. Physicians, Industry Payments for Food and Beverages, and Drug Prescribing. *JAMA* **317**, 1753 (2017).
3. Wickham, H. *ggplot2: Elegant Graphics for Data Analysis*. (Springer-Verlag New York).
4. Bauchner, H., Fontanarosa, P. B. & Flanagin, A. Conflicts of Interests, Authors, and Journals: New Challenges for a Persistent Problem. *JAMA* **320**, 2315–2318 (2018).
5. Papanicolas, I., Woskie, L. R. & Jha, A. K. Health Care Spending in the United States and Other High-Income Countries. *JAMA* **319**, 1024–1039 (2018).
6. Lo, B. & Grady, D. Payments to Physicians: Does the Amount of Money Make a Difference? *JAMA* **317**, 1719–1720 (2017).
7. McKinney, R. E., Jr & Pierce, H. H. Strategies for Addressing a Broader Definition of Conflicts of Interest. *JAMA* **317**, 1727–1728 (2017).
8. Armstrong, K. & Freiberg, A. A. Challenges and Opportunities in Disclosing Financial Interests to Patients. *JAMA* **317**, 1743–1744 (2017).
9. Barnes, B. Financial Conflicts of Interest in Continuing Medical Education: Implications and Accountability. *JAMA* **317**, 1741–1742 (2017).
10. Weil, D., Graham, M. & Fung, A. Targeting Transparency. *Science* **340**, 1410–1411 (2013).
11. Simon, P. A. *et al.* Impact of restaurant hygiene grade cards on foodborne-disease hospitalizations in Los Angeles County. *J. Environ. Health* **67**, 32–6, 56; quiz 59–60 (2005).
12. DeJong, C. & Adams Dudley, R. Reconsidering Physician–Pharmaceutical Industry Relationships. *JAMA* **317**, 1772 (2017).

13. Larkin, I. *et al.* Association Between Academic Medical Center Pharmaceutical Detailing Policies and Physician Prescribing. *JAMA* **317**, 1785–1795 (2017).
14. Schwartz, L. M. & Woloshin, S. Medical Marketing in the United States, 1997-2016. *JAMA* **321**, 80–96 (2019).
15. DeJong, C. *et al.* Pharmaceutical Industry–Sponsored Meals and Physician Prescribing Patterns for Medicare Beneficiaries. *JAMA Internal Medicine* **176**, 1114 (2016).
16. Yeh, J. S., Franklin, J. M., Avorn, J., Landon, J. & Kesselheim, A. S. Association of Industry Payments to Physicians With the Prescribing of Brand-name Statins in Massachusetts. *JAMA Intern. Med.* **176**, 763–768 (2016).
17. Perlis, R. H. & Perlis, C. S. Physician Payments from Industry Are Associated with Greater Medicare Part D Prescribing Costs. *PLoS One* **11**, e0155474 (2016).
18. Fleischman, W. *et al.* Association between payments from manufacturers of pharmaceuticals to physicians and regional prescribing: cross sectional ecological study. *BMJ* **354**, i4189 (2016).
19. Sharma, M., Vadhariya, A., Johnson, M. L., Marcum, Z. A. & Holmes, H. M. Association between industry payments and prescribing costly medications: an observational study using open payments and medicare part D data. *BMC Health Serv. Res.* **18**, 236 (2018).
20. Zezza, M. A. & Bachhuber, M. A. Payments from drug companies to physicians are associated with higher volume and more expensive opioid analgesic prescribing. *PLoS One* **13**, e0209383 (2018).
21. Fickweiler, F., Fickweiler, W. & Urbach, E. Interactions between physicians and the pharmaceutical industry generally and sales representatives specifically and their association with physicians' attitudes and prescribing habits: a systematic review. *BMJ Open* **7**, e016408 (2017).

22. Carpenter, D. & Joffe, S. A unique researcher identifier for the Physician Payments Sunshine Act. *JAMA* **305**, 2007–2008 (2011).

23. Pham-Kanter, G., Mello, M. M., Lehmann, L. S., Campbell, E. G. & Carpenter, D. Public Awareness of and Contact With Physicians Who Receive Industry Payments: A National Survey. *J. Gen. Intern. Med.* **32**, 767–774 (2017).

24. Consult, M. *How Patients Will Use Physician Payment Data - Morning Consult*. (2014).

25. Rose, S. L. *et al.* Patient responses to physician disclosures of industry conflicts of interest: A randomized field experiment. *Organizational Behavior and Human Decision Processes* (2019). doi:10.1016/j.obhdp.2019.03.005

26. Kanter, G. P., Carpenter, D., Lehmann, L. S. & Mello, M. M. US Nationwide Disclosure of Industry Payments and Public Trust in Physicians. *JAMA Netw Open* **2**, e191947 (2019).

REVIEWERS' COMMENTS:

Reviewer #1 (Remarks to the Author):

I appreciate the authors careful and detailed response to the first round of reviews. I think the paper is significantly strengthened---particularly with regards to limiting the casual claims and cautioning the implications of the results. I have remaining concerns about over interpreting associations, especially with regards to the political results which I think requires a more detailed policy economy understanding of mechanisms, but I think that this debate should happen in the open after the article is published. Wonderful job.

Reviewer #2 (Remarks to the Author):

No further comments - all my questions and critiques have been addressed.

REVIEWERS' COMMENTS:

Reviewer #1 (Remarks to the Author):

I appreciate the authors careful and detailed response to the first round of reviews. I think the paper is significantly strengthened---particularly with regards to limiting the casual claims and cautioning the implications of the results. I have remaining concerns about over interpreting associations, especially with regards to the political results which I think requires a more detailed policy economy understanding of mechanisms, but I think that this debate should happen in the open after the article is published. Wonderful job.

Thank you for the helpful comments throughout this revision. We have revised the abstract per your suggestion.

Reviewer #2 (Remarks to the Author):

No further comments - all my questions and critiques have been addressed.

Thank you for the helpful comments throughout this revision.

Comparison between previous and current Figure 1.

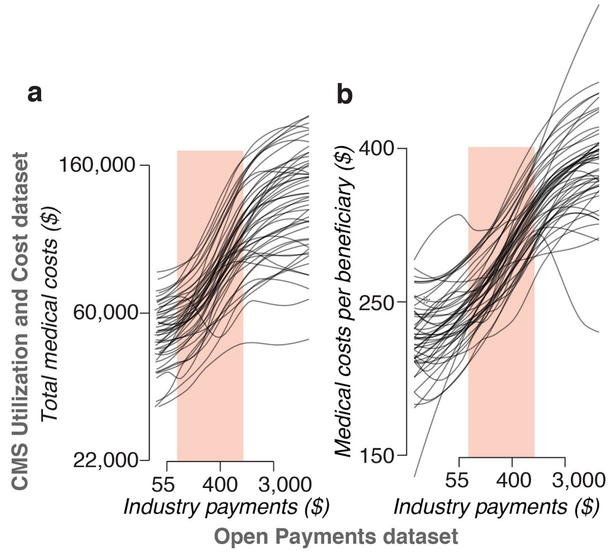

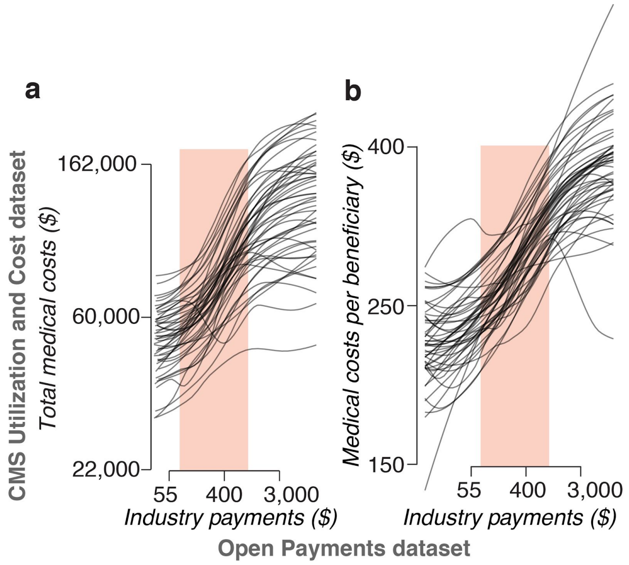

Top Figure 1 submitted Revision 1 (June 2019).

Bottom Figure 1 submitted with this Final version of the manuscript (August 2019).

Comparison between previous and current Figure 2.

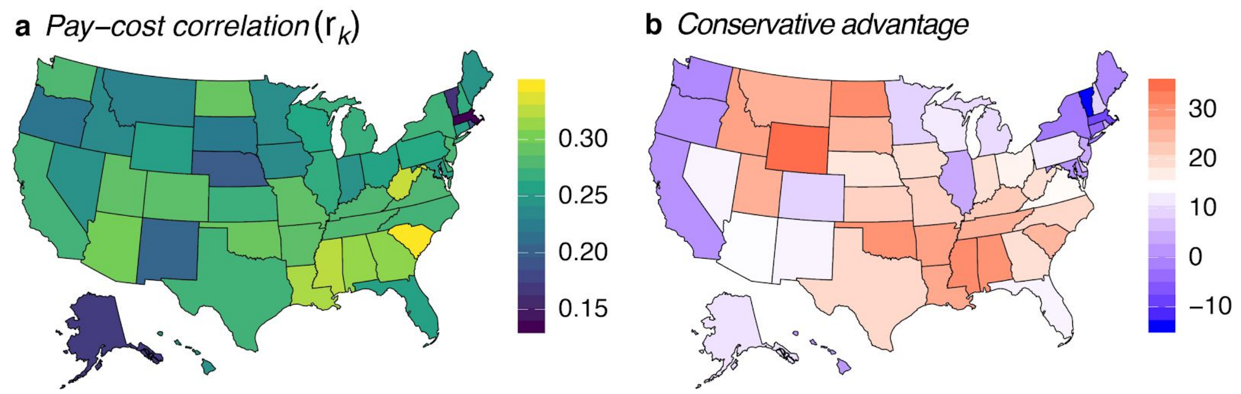

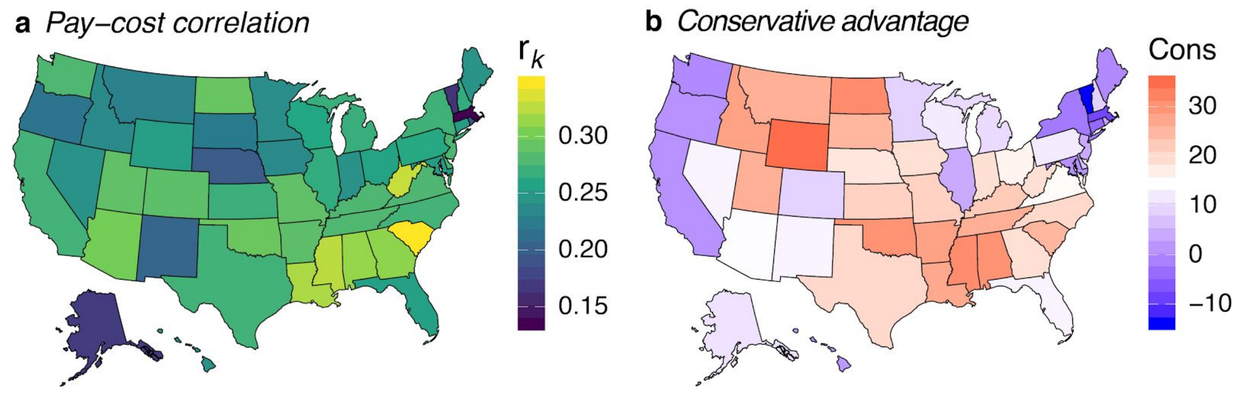

Top Figure 2 submitted Revision 1 (June 2019).

Bottom Figure 2 submitted with this Final version of the manuscript (August 2019).

Comparison between previous and current Figure 3.

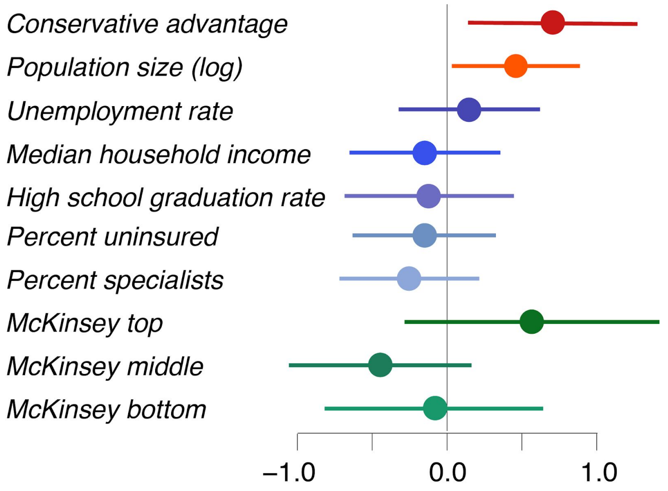

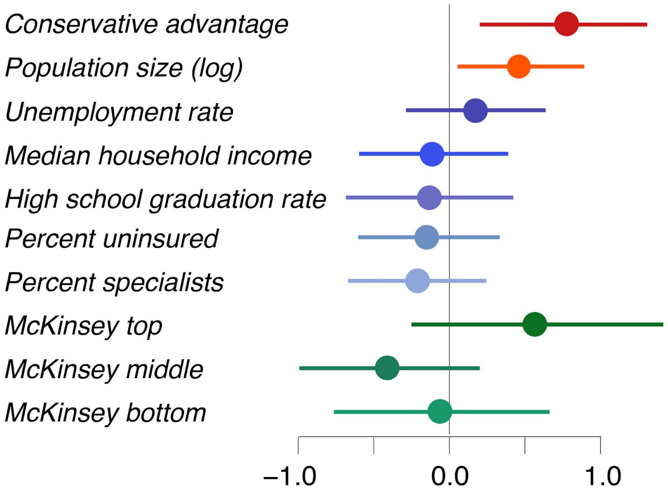

Top Figure 3 submitted Revision 1 (June 2019).

Bottom Figure 3 submitted with this Final version of the manuscript (August 2019).
